# Assessing the efficacy and safety of magnesium sulfate for management of autonomic nervous system dysregulation in Vietnamese children with severe hand foot and mouth disease

**DOI:** 10.1186/s12879-019-4356-x

**Published:** 2019-08-22

**Authors:** Qui Tu Phan, Lam Khanh Phung, Khanh Huu Truong, Trieu Trung Huynh, Giang Thanh Phạm, Bich Ngọc Nguyen, Quyen Thanh Tran, Vuong Ngoc Thien Huynh, Tien Thi My Nguyen, Thoa Phan Kim Le, Nhan Nguyen Thanh Le, Saraswathy Sabanathan, H. Rogier van Doorn, Tan Van Le, Toan Duc Nguyen, Laura Merson, Dung Thi Phuong Nguyen, Ronald Geskus, Hung Thanh Nguyen, Chau Van Vinh Nguyen, Bridget Wills

**Affiliations:** 1 Hospital for Tropical Diseases, 764 Vo Van Kiet, District 5, Ho Chi Minh City, Vietnam; 2 Oxford University Clinical Research Unit, Hospital for Tropical Diseases, 764 Vo Van Kiet, Quan 5, Ho Chi Minh City, Vietnam; 3 Children’s Hospital Number 1, 341 Sư Vạn Hạnh, District 10, Ho Chi Minh City, Vietnam; 4 Present Address: Paediatric Neurology Department, Cambridge University Hospital NHS Trust, Cambridge, UK; 5 Centre for Tropical Medicine and Global Health, Nuffield Department of Clinical Medicine, Oxford University, Oxford, UK; 6 Present Address: Infectious Diseases Data Observatory, Centre for Tropical Medicine and Global Health, Oxford University, Oxford, UK

**Keywords:** Hand foot and mouth disease, Brainstem encephalitis, Autonomic nervous system dysregulation, Hypertension, Magnesium sulfate, Clinical trial

## Abstract

**Background:**

Brainstem encephalitis is a serious complication of hand foot and mouth disease (HFMD) in children. Autonomic nervous system (ANS) dysregulation and hypertension may occur, sometimes progressing to cardiopulmonary failure and death. Vietnamese national guidelines recommend use of milrinone if ANS dysregulation with Stage 2 hypertension develops. We wished to investigate whether magnesium sulfate (MgSO_4_) improved outcomes in children with HFMD if used earlier in the evolution of the ANS dysregulation (Stage 1 hypertension).

**Methods:**

During a regional epidemic we conducted a randomized, double-blind, placebo-controlled trial of MgSO_4_ in children with HFMD, ANS dysregulation and Stage 1 hypertension, at the Hospital for Tropical Diseases in Ho Chi Minh city. Study participants received an infusion of MgSO_4_ or matched placebo for 72 h. We also reviewed data from non-trial HFMD patients in whom milrinone failed to control hypertension, some of whom received MgSO_4_ as second line therapy. The primary outcome for both analyses was a composite of disease progression within 72 h - addition of milrinone (trial participants only), need for ventilation, shock, or death.

**Results:**

Between June 2014 and September 2016, 14 and 12 participants received MgSO_4_ or placebo respectively, before the trial was stopped due to futility. Among 45 non-trial cases with poorly controlled hypertension despite high-dose milrinone, 33 received MgSO_4_ while 12 did not. There were no statistically significant differences in the composite outcome between the MgSO_4_ and the placebo/control groups in either study (adjusted relative risk (95%CI) of [6/14 (43%) vs. 6/12 (50%)], 0.84 (0.37, 1.92), *p* = 0.682 in the trial and [1/33 (3%) vs. 2/12 (17%)], 0.16 (0.01, 1.79), *p* = 0.132 in the observational cohort). The incidence of adverse events was similar between the groups. Potentially toxic magnesium levels occurred very rarely with the infusion regime used.

**Conclusion:**

Although we could not demonstrate efficacy in these studies, there were no safety signals associated with use of 30-50 mg/kg/hr. MgSO_4_ in severe HFMD. Intermittent outbreaks of HFMD are likely to continue across the region, and an adequately powered trial is still needed to evaluate use of MgSO_4_ in controlling hypertension in severe HFMD, potentially involving a higher dose regimen.

**Trial registration:**

ClinicalTrials.gov Identifier: NCT01940250 (Registered 22 AUG 2013).

**Trial sponsor**: University of Oxford

**Electronic supplementary material:**

The online version of this article (10.1186/s12879-019-4356-x) contains supplementary material, which is available to authorized users.

## Background

Over the last 15 years hand, foot and mouth disease (HFMD) has become an increasingly important cause of paediatric morbidity and mortality in Vietnam, and has placed a huge burden on healthcare services across the Asia-Pacific region [1]. During outbreaks thousands of young children can be affected, and while HFMD is typically mild and self-limited, severe complications do occur, albeit rarely. Most notably, brainstem encephalitis may develop, presenting with autonomic nervous system (ANS) dysregulation around the third or fourth day of fever, sometimes with severe hypertension that may progress to cause cardiopulmonary failure [2]. Typically, the period of ANS dysregulation lasts for 48-72 h, although in severe cases progression may be rapid, with death occurring within a few hours.

Management of ANS dysregulation in children presents particular challenges. A phosphodiesterase inhibitor, milrinone, has become the recommended therapy for severe HFMD, based on findings from two small studies, one retrospective, involving 65 participants overall, that reported benefit with milrinone in patients with cardiopulmonary failure [3, 4]. For children with HFMD and ANS dysregulation, current Vietnamese Ministry of Health (MoH) guidelines recommend close observation without anti-hypertensive therapy when the systolic blood pressure (SBP) remains between the 95th percentile and 5 mmHg above the 99th percentile for age (i.e Stage 1 hypertension [5]), but to intervene promptly (ideally within 1 h) with milrinone when the SBP exceeds the 99th centile for age plus 5 mmHg, (i.e. Stage 2 hypertension [5]). However, despite use of high dose milrinone in accordance with these recommendations, some children continue to deteriorate, rapidly requiring ventilatory support and/or haemofiltration [6]. Safety data regarding milrinone use in children are limited, although associations with tachyarrhythmias and acute renal failure have been documented [7, 8].

Tetanus is another disease in which ANS dysregulation with severe hypertension occurs. Extrapolating from research in adults with tetanus [9–11], and from experience in rare conditions such as phaeochromocytoma [12, 13], magnesium sulfate (MgSO_4_) has become the drug of choice to control ANS dysregulation in neonates with tetanus managed on the Paediatric Intensive Care Unit (PICU) at the Hospital for Tropical Diseases (HTD) in Ho Chi Minh City, using a regimen of 30–50 mg/kg per hour for up to 7 days titrated according to response [14]. The physiological rationale underlying use of MgSO_4_ in these circumstances relies on several properties of magnesium (Mg) ions: they compete with calcium (Ca) ions for receptors on vascular smooth muscle cells and can influence BP by modulating vascular tone [15]; Mg has an important role in the classical pathway of nitric oxide (NO) release, with changes in extracellular Mg content modifying production and release of NO and thereby altering arterial tone [16]; Mg also decreases the release of catecholamines after sympathetic stimulation [17]. MgSO_4_ is cheap, readily available and easily neutralised, and is therefore generally considered to be safe although little formal safety data is available for children [18–20]. Given the experience with neonatal tetanus, when a major outbreak of HFMD commenced across the region in 2011 and use of high-dose milrinone proved inadequate to control ANS dysregulation in several cases, MgSO_4_ was adopted at HTD as second line treatment with promising initial results [21].

We hypothesized that intervention with MgSO_4_ early, when hypertension due to ANS dysregulation first becomes apparent, might control cardiovascular instability more effectively and prevent progression to severe disease. In June 2014 we commenced a randomized, double-blind, placebo-controlled intervention trial to evaluate the efficacy and safety of MgSO_4_ in 190 children with HFMD, ANS dysregulation and Stage 1 hypertension [19]. However, over the next 2 years the number of HFMD cases seen across Vietnam declined dramatically, and by the end of 2016 the trial was stopped on the grounds of futility.

Over the 18-month period needed to obtain funding and the necessary ethical approvals to commence the trial, open-label MgSO_4_ was often used to treat severe cases. We therefore reviewed the hospital files of all non-trial severe HFMD cases managed on PICU from 2011 onwards to identify those who had received open-label MgSO_4_, aiming to gather additional data on the efficacy and safety of MgSO_4_ in severe HFMD, in the knowledge that major outbreaks of HFMD are likely to continue across the region in the coming years.

## Methods

### The clinical trial

This was a randomized, double-blind, placebo-controlled trial of intravenous MgSO_4_ versus placebo in Vietnamese children with a clinical diagnosis of Grade 3 HFMD (according to guidelines issued by the Vietnamese MoH [22]) and signs of ANS dysregulation including systemic hypertension. The study was approved by the ethics committees of HTD and the Vietnamese MoH, as well as the Oxford University Tropical Research Ethics Committee, and was carried out in strict compliance with all ICH-GCP guidelines and recommendations. The trial was registered with ClinicalTrials.gov (NCT01940250), and the full protocol has been published [23].

Essential details of the general study methodology are presented in Additional file 1: Appendix A, and the Vietnamese grading system for HFMD is summarised in Additional file 2: Table S1. In brief, patients aged 6 months to 15 years admitted to PICU at HTD with a clinical diagnosis of Grade 3 HFMD were eligible for enrolment if the BP, measured invasively, was sustained above the cut-off for Stage 1 hypertension (i.e. the 95th centile for age, gender and length [24]) for at least 30 min while the child was not distressed, and the individual exhibited at least one other criterion of ANS dysregulation. Children presenting with Stage 2 hypertension were also eligible for enrolment provided they had no evidence of acute target organ damage, with the proviso that study treatment must commence within 30 min of admission and that milrinone should be added within a further 30 min if there was no improvement – ie the total time to commencing milrinone should be 1 h if the child did not improve (see Additional file 1: Appendix A for further information). An explanation of the Vietnamese guidelines for treatment of severe HFMD was given to the parent/guardian, together with specific information on the proposal to intervene with either MgSO4 or placebo when their child would not usually receive specific therapy for hypertension and details of the systems in place to commence specific treatment with milrinone within 1 h should this be necessary. Following written informed consent by the parent/guardian, study participants were randomly allocated in a 1:1 ratio to receive a loading dose of 50 mg/kg of either 10% MgSO_4_ (Fresenius Kabi, Germany) or visually matched placebo. The study drug was given as a continuous infusion over 20 min followed by a maintenance infusion of 30–50 mg/kg/hr. for 72 h according to response, aiming for plasma total Mg levels between 1.8 and 2.5 mmol/l in the treatment arm. Recognizing that having a child in ICU is extremely stressful, detailed information on the study protocol was presented to the family a second time within 12–24 h of enrolment, in order to be sure that they had fully understood the information presented earlier. At this time the option to withdraw from the study was again clearly articulated to the parent/guardian.

All staff involved in clinical care were blind to the treatment allocation, and Mg levels were monitored and adjusted by independent doctors from another clinical facility; these doctors focused particularly on safety, especially given that children in the active arm were receiving an antihypertensive agent at an earlier stage than usual. Detailed clinical assessments were performed daily, with vital signs documented at least hourly for the first 72 h and full biochemical profiles and ECGs performed at least once daily. Catecholamine levels, specifically adrenaline and noradrenaline, were measured on plasma and urine specimens collected daily for 3 days after study enrolment between 8 and 10 am. All clinical and laboratory adverse events were graded following the CTCAE Version 4.03 guidelines [25], modified for children (Additional file 1: Appendix A.7, A.8).

The primary endpoint was a composite of disease progression defined as occurrence of any of the following within 72 h of commencing the study drug: pre-specified BP criteria necessitating addition of milrinone in accordance with MoH guidelines for HFMD with Stage 2 hypertension; ventilation; shock; or death. Secondary outcomes included the time to requirement for milrinone, the area under the curve (AUC) for heart rate (HR), systolic blood pressure (SBP), and mean arterial pressure (MAP) above the Stage 1 hypertension level during the first 72 h, the duration of hospitalization, and neurodevelopmental status assessed 6 months after discharge [26].

### The observational cohort

Using HTD’s electronic patient database, we identified the hospital files of all children with HFMD who received milrinone between January 2011 and December 2015. From these files we identified cases where the BP remained above the Stage 2 hypertension level despite high dose milrinone (0.6–0.7 μg/kg/minute) but without cardiovascular decompensation at that time (see Additional file 1: Appendix B for details). We wished to compare responses in terms of hemodynamic stability and severe outcomes in patients who fulfilled these criteria and received MgSO_4_, with similar cases who achieved the same basic cardiovascular severity level but for whom MgSO_4_ was not used. All available data on clinical progress, management and outcomes were extracted from the hospital files to a special case report form (CRF) and entered into an electronic database. The MgSO_4_ regimen used in the exposed patient group was nominally the same as that used in the trial. The primary outcome was a composite including development of shock, respiratory compromise requiring ventilation, or death, occurring within the first 72 h from time zero – i.e. the time when MgSO_4_ was actually introduced (exposed group), or might theoretically have been introduced based on the individual’s cardiovascular status (control group). Secondary outcomes included the AUC for SBP and MAP above the Stage 1 hypertension level over 24 h from time zero, (since subsequent blood pressure monitoring was less detailed than in the trial), and the duration of hospitalization.

### Statistical analysis

Summary statistics are absolute count and percentage for categorical variables and median (range) for continuous data. For the trial, in view of the small number of patients enrolled we only performed the intention-to-treat analysis. For both the trial and the observational study, between-group comparisons for the primary outcome of disease progression were based on log-binomial regression models (generalized linear models for binomial outcome and using log link function) with adjustment for age and day of illness at study entry/time zero.

For the trial secondary outcomes, the time from commencement of study drug to addition of milrinone was compared using the log-rank test and a Cox proportional hazards model with adjustment for baseline SBP and day of illness at study entry. Patients who never received milrinone were censored at 72 h. Between-group comparisons of the AUCs for the various cardiovascular parameters were based on linear regression models with adjustment for the baseline value of the variable of interest. As AUCs for SBP and MAP were skewed to the right, we log10-transformed these variables before applying regression analysis. For the neurodevelopmental assessments, Z scores for each domain were derived for each individual using data from healthy Vietnamese children matched by age group [26]. We then calculated mean differences in these Z scores between the intervention groups using linear regression and adjusting for age, sex and maternal education level. For comparison of the catecholamine profiles, we used a linear mixed effects regression model that accounted for the evolution of the responses in the two groups.

In the observational study, the group of children who did not receive MgSO_4_ were generally hospitalised during the early period of the outbreak, thus forming a historical control group for those who received MgSO_4_ later_._ For the exposed group, decision-making regarding when to commence MgSO_4_ appeared to be influenced by the patient’s age, the rapidity of the deterioration, and the experience of the clinician, in addition to the specific BP criteria outlined above and in Additional file 1: Appendix B. We therefore developed a logistic regression model for initiation of MgSO_4_ for each time point at which SBP was measured in the exposed group. We then used this model to impute a time at which MgSO_4_ could have been initiated for each set of observations for the control patients. We produced 20 imputed datasets and performed comparisons similar to those described above for the clinical trial on each dataset, finally combining the results using Rubin’s rule [27] and a modified rule that accounted for differences in size between imputed datasets. Detailed information describing these procedures is provided in Additional file 1: Appendix B, Additional file 3: Tables S2, Additional file 4: Table S3 and Additional file 5: Table S4.

## Results

### The clinical trial

**Fig. 1:**
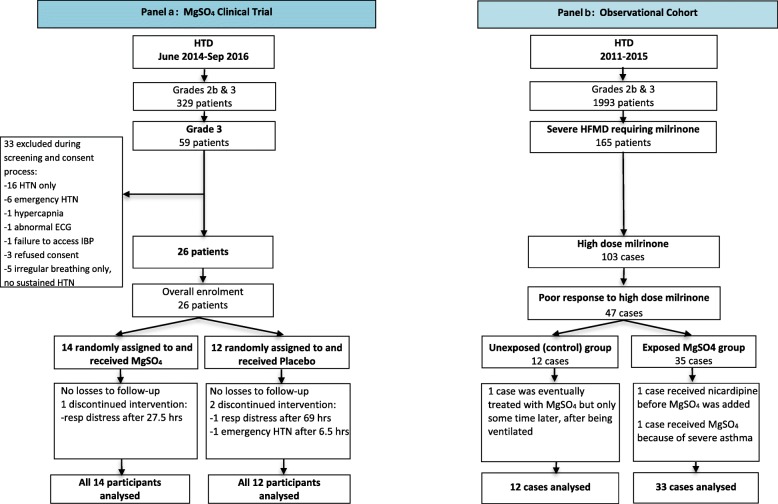
Patient recruitment and study group allocation in the clinical trial (Panel **a**) and the observational cohort (Panel **b**). Abbreviations: HTD – Hospital for Tropical Diseases, HTN – hypertension, IBP- Invasive Blood Pressure

Between June 2014 and September 2016, 59 children with Grade 3 HFMD and signs of ANS dysregulation underwent screening for potential inclusion in the trial, of whom 26 (MgSO_4_ = 14, Placebo = 12) were enrolled following written consent by a parent or guardian (Fig. 1, Panel a). Compliance with all aspects of the study protocol was excellent, with only a small number of minor protocol deviations. All trial participants received the designated study drug according to the randomization code, and 23/26 cases completed the full 72-h infusion as per protocol. The study drug was unblinded in 3 cases. One child in the placebo group developed emergency hypertension (Additional file 1: Appendix A.3); after unblinding showed that the child was allocated to the placebo arm, open label MgSO_4_ was commenced, following which the BP settled without additional therapy. The two other children developed severe respiratory distress requiring ventilation; one child was in the placebo group, with Mg/total calcium (Ca) levels within the normal range, while the other was in the active intervention arm, with plasma Mg and Ca levels of 2.3 and 1.9 mmol/l respectively.

Clinical and laboratory characteristics were similar at baseline between the MgSO_4_ and placebo groups (Table 1), except that the proportion of children with Stage 2 hypertension was considerably greater in the placebo group (8/12, 67%) than in the MgSO_4_ group (3/14, 21%). Enteroviruses were detected by generic RT-PCR [28] in 18/26 (70%) of the cases overall, which is within the same range as previous reports for clinically diagnosed HFMD [29, 30], with EV-A71 confirmed by specific RT-PCR [31] in 9/14 (64%) MgSO_4_ recipients and 4/12 (33%) placebo recipients.

**Table 1:** Baseline information for the clinical trial population

	Placebo (*N* = 12)	MgSO_4_ (*N* = 14)
Demographic and clinical features		
Age (months)	21 (7, 57)	24 (9, 73)
Sex (female)	6 (50)	9 (64)
Weight (kg)	11 (7, 19)	11 (7, 20)
Illness day at enrolment	3.5 (1.0, 6.0)	3.0 (2.0, 5.0)
Fever (> 37.5 °C)	12 (100)	13 (93)
Mouth ulcers present	10 (83)	11 (79)
Skin lesions present	9 (75)	11 (79)
Skin ANS features^a^	2 (17)	1 (7)
Tachycardia^b^	2 (17)	1 (7)
SBP (mmHg)	116 (105, 129)	113 (101, 134)
DBP (mmHg)	61 (52, 73)	59 (47, 73)
Stage of hypertension		
Stage1	4 (33)	11 (79)
Stage2	8 (67)	3 (21)
Tachypnea	5 (42)	6 (43)
Irregular breathing	7 (58)	5 (36)
Respiratory retractions	1 (8)	3 (21)
Irritability	4 (33)	1 (7)
Neurological abnormalities^c^	3 (25)	0 (0)
Laboratory investigations		
Arterial blood gases		
- pH	7.44 (7.40, 7.59)	7.41 (7.36, 7.48)
- pCO_2_ (mmHg)	29.8 (18.6, 35.7)	33.3 (29.4, 40.1)
Hb (g/dl)	11.8 (8.8, 15.2)	11.8 (6.4, 14.3)
WBC (× 10^9^/l)	10.2 (4.6, 15.5)	13.6 (5.5, 26.5)
CK-MB (IU/l)	23.4 (10.4, 64.8)	25.4 (13.8, 48.1)
Troponin I (pg/ml)	5 (0, 20)	5 (0, 27)
Mg (mmol/l)	0.85 (0.70, 0.99)	0.88 (0.78, 0.98)
Ca (mmol/l)	2.28 (1.69, 2.52)	2.31 (1.95, 2.42)
Na (mmol/l)	131 (126, 134)	131 (125, 136)
Creatinine (μmol/l)	26 (17, 52)	27 (18, 42)
Blood sugar (mmol/l)	5.4 (4.0, 7.6)	5.7 (5.0, 8.1)
Lab-confirmed EV infection	7 (58)	11 (78)
EV-A71 positive	4 (33)	9 (64)
Other EV positive^d^	3 (25)	2 (14)

Summary statistic is absolute count (%) for categorical variables and median (range) for continuous data

*SBP* Systolic blood pressure, *DBP* Diastolic blood pressure, *EV-A71* Enterovirus A71, *EV* Enterovirus

^a^Skin manifestations of autonomic nervous system (ANS) dysregulation

^b^Tachycardia: Heart rate sustained > 150 beats/min, adjusted down by 10 for each 1 degree of fever above 37.0

^c^Myoclonic jerks were noted in 2 cases, while limb tremor/ataxia and nystagmus were present in 1 case, all in the placebo group

^d^Includes 3 cases of Coxsackievirus (CV) A16, 1 case of CV A10, 1 case of CV –C, all other cases were negative

**Fig. 2:**
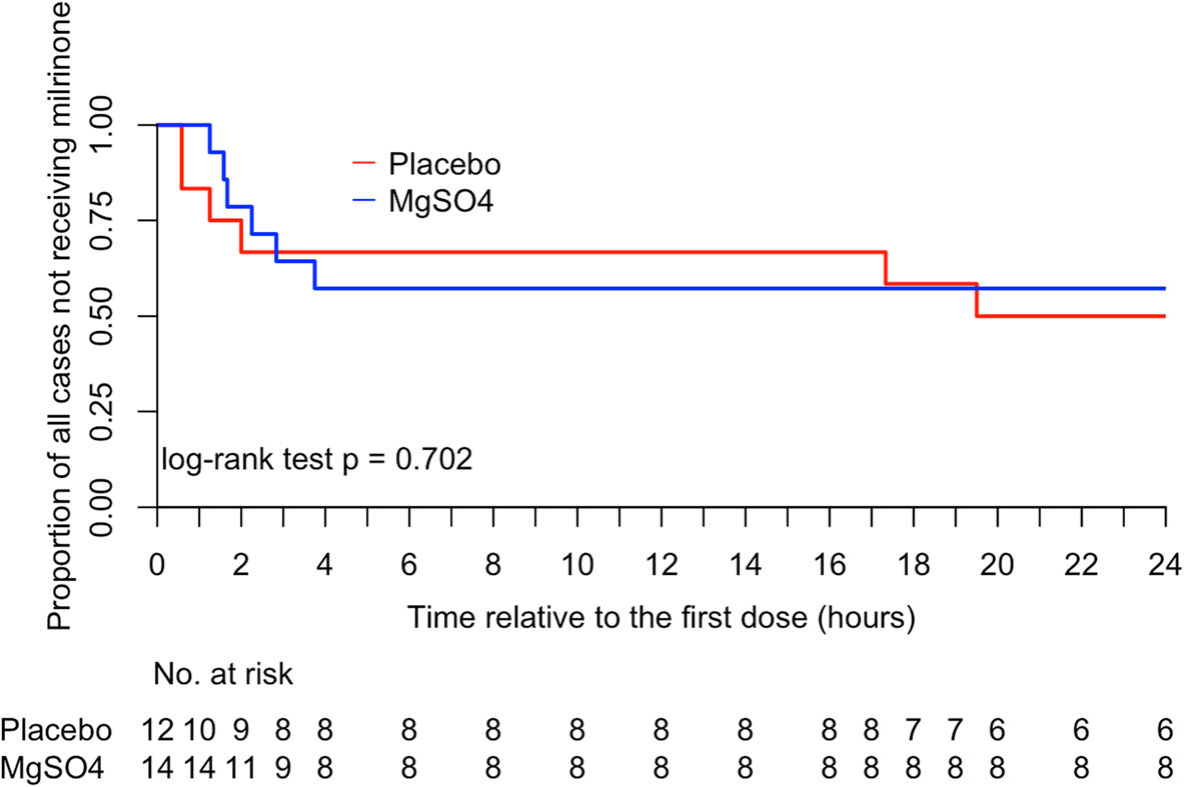
Kaplan-Meier curves showing the time from baseline to addition of milrinone in the clinical trial

No patient died or developed shock, while 6/12 (50%) cases in the placebo group required milrinone compared to 6/14 (43%) cases in the MgSO_4_ group. Two children who received milrinone also required ventilation, one in each treatment group. There was no significant difference in the composite primary outcome between the two groups, adjusted relative risk (95%CI) 0.84 (0.37, 1.92), *p* = 0.682 (Table 2). There was also no significant difference in the time from study drug initiation to addition of milrinone between the treatment groups (Table 2, Panel A and Fig. 2). Evaluation of the AUCs for the various cardiovascular parameters showed each AUC to be slightly lower in the MgSO_4_ group, but there were no statistically significant differences (Table 3).

**Table 2:** Primary and secondary endpoints observed in the different treatment groups in both studies^a^

	Panel A: Clinical Trial				Panel B: Observational Cohort			
	Placebo (*N* = 12)	MgSO_4_ (*N* = 14)	Estimated effect (95% CI)	*p*-value	Control (*N* = 12)	MgSO_4_ (*N* = 33)	Estimated effect (95% CI)	*p*-value
Primary endpoint^b^	6 (50%)	6 (43%)	0.84 (0.37, 1.92)	0.682	2 (17%)	1 (3%)	0.16 (0.01, 1.79)	0.132
Requirement for ventilation	1 (8%)	1 (7%)	–		2 (17%)	1 (3%)	0.16 (0.01, 1.79)	0.132
Requirement for milrinone	6 (50%)	6 (43%)	0.80 (0.25, 2.55)	0.706	–	–	–	
Time to start of milrinone^c^	1.6 (0.6, 19.5)	2.0 (1.2, 3.8)			–	–	–	
Length of hospitalization^c^	5 (5, 6)	5 (5, 6)	0.12 (−1.55, 1.79)	0.889	9 (8, 13)	9 (7, 10)	−1.65 (−3.79, 0.49)	0.117

The estimated effect for the primary endpoint and for requirement for ventilation is the relative risk, while for length of hospitalization it is the mean difference, and for requirement for milrinone it is the hazard ratio

Results are based on log-binomial regression (primary endpoint, requirement for ventilation), linear regression (length of hospitalization), Cox regression (requirement for milrinone). For those patients who never received milrinone the nominal start time was fixed at 72 h. All analyses were adjusted for age and illness day (except for Cox regression analysis which was adjusted for illness day and systolic blood pressure at baseline)

^a^Panel A for the clinical trial and Panel B for the observational cohort

^b^For the clinical trial the primary endpoint was a composite outcome of death or shock or requirement for ventilation or need for milrinone, while for the observational cohort it comprised death or shock or requirement for ventilation (since all study participants were already on milrinone)

^c^Length of hospitalization and time to start of milrinone are summarized in terms of median (range) values. Length of hospitalization is the number of days from study enrolment to hospital discharge. Time to start of milrinone is the number of hours from initiation of study drug to addition of milrinone, and is only described for patients who received milrinone

**Table 3:** Effect of MgSO_4_ on selected hemodynamic parameters in the clinical trial and the observational cohort

	Placebo / Control^a^	MgSO_4_	Mean difference (95% CI)	*p*-value
Clinical Trial (for 72 h from study drug initiation)	(*N* = 12)	(*N* = 14)		
AUC of HR (beats/min x hr)				
Median (range)	9728 (8820, 10,983)	9021 (8156, 11,008)		
Mean (sd)	9816 (744)	9296 (931)	− 581 (− 1264, 102)	0.095
Log-10 AUC of SBP above stage 1 HTN (log10(mmHg x hr))				
Median (range)	2.52 (1.80, 3.22)	2.40 (1.48, 3.09)		
Mean (sd)	2.46 (0.39)	2.36 (0.47)	−0.07 (− 0.40, 0.27)	0.701
Log-10 AUC of MAP above stage 1 HTN (log10(mmHg x hr))				
Median (range)	2.16 (1.00, 3.04)	2.13 (0.00, 2.69)		
Mean (sd)	2.09 (0.63)	2.03 (0.68)	0.05 (−0.45, 0.55)	0.850
Observational cohort (for 24 h from study drug initiation)	(*N* = 12)	(*N* = 33)		
Log-10 AUC of SBP above stage 1 HTN (mmHg x hr)^b^				
Median (range)	2.47 (1.82, 2.87)	2.50 (1.54, 2.83)		
Mean (sd)	2.44 (0.31)	2.42 (0.33)	−0.05 (−0.26, 0.15)	0.604
Log-10 AUC of MAP above stage 1 HTN (mmHg x hr)^b^				
Median (range)	1.54 (0.00, 2.74)	1.62 (0.00, 2.56)		
Mean (sd)	1.62 (0.72)	1.54 (0.57)	−0.17 (−0.54, 0.19)	0.354

Note: In the clinical trial the study drug was commenced at Stage 1 hypertension without milrinone, while in the observational cohort MgSO_4_ was commenced at Stage 2 hypertension when the patients were already on high dose milrinone

Comparisons for AUCs of HR, SBP and MAP were based on linear regression with adjustment for their corresponding values at baseline

*HR* Heart rate, *SBP* Systolic blood pressure, *MAP* Mean arterial pressure, *AUC* Area under the curve, *HTN* Hypertension, *sd* standard deviation

^a^Placebo Group in the clinical trial and Control Group in the observational cohort

^b^For the control group, results are based on multiple imputation of the time that MgSO_4_ would have commenced. The imputation model is a logistic regression model based on: a) the difference between the current SBP and the age-dependent cut-off for Stage 2 hypertension; b) the difference between the current SBP and the previous SBP value; and c) the current dose of milrinone. The descriptions were averaged across imputation datasets while the comparisons were based on Rubin’s rule (a modified rule to take into account difference in size between imputed datasets gave similar results). Detailed information describing this methodology can be found in the Additional file 1, Additional file 3: Table S2, Additional file 4: Table S3 and Additional file 5: Table S4

Plasma catecholamine levels were generally elevated or at the upper end of the expected normal ranges for children at study enrolment (Additional file 6: Figure S1), falling progressively over the 3 days period of observation. However, there were no significant differences in the overall profiles between the study arms (*p* = 0.609 and *p* = 0.997, respectively, for plasma adrenaline and noradrenaline levels). Urine catecholamine levels were also increased at enrolment, fell progressively over time, and were generally lower in the MgSO_4_ group compared to the placebo group at each timepoint, but with no statistically significant differences overall (*p* = 0.545 and *p* = 0.589, respectively, for urine adrenaline and noradrenaline levels).

A total of 21 clinical adverse events (AEs) occurred in 10 patients in the placebo group (83%) compared to 22 events in 10 children in the MgSO_4_ group (71%) (Table 4). Serious clinical AEs occurred in 5 patients overall. Respiratory distress developed in 2 patients in the placebo group and 3 in the MgSO_4_ group, with one patient in each group requiring ventilation. In no case were the AEs or SAEs associated with Mg levels in excess of 3 mmol/l (maximum recorded value, 2.72 mmol/l), the usual threshold of concern for respiratory or CNS depressant effects of hypermagnesaemia. Laboratory abnormalities were common but were observed with similar frequencies in the two study arms.

**Table 4:** Adverse events observed in the two treatment arms in the clinical trial

	Number of patients with event	
	Placebo (*n* = 12)	MgSO_4_ (*n* = 14)
Clinical events		
All adverse events^a^	21 / 10 (83)	22 / 10 (71)
Fever	6	7
Diminished deep tendon reflexes	2	3
Coma / lethargy / irritability	2	1
Respiratory problems	3	4
Reduced urine output (< 1 ml/kg/hr for 4 h)	4	5
Other^b,c^	4	2
Serious adverse events (Grades 3 and 4)^a^	3 / 2 (17)	3 / 3 (21)
Coma	1	0
Respiratory distress	2	3
Laboratory events		
All adverse events^a^	46 / 12 (100)	51 / 13 (93)
Respiratory Acidosis	0	3
Respiratory Alkalosis	8	6
Abnormal CK-MB	7	9
Abnormal Creatinine	2	2
Abnormal Hb	4	4
Hyperkalemia	2	0
Hypokalemia	5	2
Hyponatremia	9	9
Abnormal Troponin I	5	5
Serious adverse events (Grades 3 and 4)^a^	16 / 8 (67)	21 / 10 (71)
Respiratory Acidosis	0	1
Respiratory Alkalosis^d^	3	6
Abnormal CK-MB	1	0
Abnormal Hb	1	0
Hypokalemia	1	1
Hyponatremia^e^	5	7
Abnormal Troponin I	5	4

Adverse events were graded using the CTCAE Version 4.03 system, modified for children

^a^Numbers are total events / events grouped by patient (% of patients in the group with any events)

^b^Placebo group: 2 with generalised erythema, 1 diarrhoea, 1 measles

^c^MgSO_4_ group: 1 case each of vomiting and myoclonic jerks

^d^In all cases respiratory alkalosis occurred in spontaneously breathing patients

^e^Severe hyponatremia developed in patients not receiving concurrent intravenous fluids

All participants were well enough for discharge by study day 14, although 3 children had persisting clinically apparent neurological problems: two with generalized weakness and one with 9th and 11th cranial nerve palsies. However, all three had recovered by the 6-month follow-up visit, at which time all study participants had normal neurological and neurodevelopmental assessments (see Additional file 3: Table S2).

### The observational cohort

From January 2011 to December 2015, 165 children with severe HFMD were treated with milrinone (Fig. 1, Panel b). Among the 103 children who received high dose milrinone for Stage 2 hypertension, 45 cases where the BP remained poorly controlled were selected for detailed study; 33 received MgSO_4_ (the exposed group) while 12 did not (the control group). The baseline characteristics were similar between the two groups, except that the children in the MgSO_4_ group were significantly older than the controls and EV-A71 was identified less frequently in the MgSO_4_ recipients, (23/33 (70%) compared to 12/12 (100%)) (Table 5). Of note, 11 of the 12 children in the control group were admitted in the early stage of the outbreak when EV-A71 was known to be prevalent, whereas later the pattern of serotypes in circulation became more diverse [32].

**Table 5:** Clinical and laboratory features in the observational cohort for the MgSO_4_ exposed and control groups^a^

	MgSO_4_ (*N* = 33)	Control (*N* = 12)
Demographic and clinical features^b^		
Age (months)	36 (12, 153)	15 (6, 45)
Sex (female)	7 (21)	6 (50)
Weight (kg)	18 (9, 55)	10 (7, 20)
Illness day at T = 0	4 (2, 8)	4 (1, 7)
Fever (> 37.5^0^ C)	26 (79)	11 (92)
Skin ANS features^c^	1 (3)	0 (0)
Tachycardia^d^	7 (21)	2 (17)
Systolic BP at T = 0	140 (122, 200)	134 (119, 168)
Diastolic BP at T = 0	70 (55, 110)	64 (54, 88)
Tachypnea for age	16 (55)	9 (75)
Irregular breathing	3 (12)	2 (18)
Neurological abnormalities^e^	1 (3)	0 (0)
Laboratory investigations^b^		
Hb (g/dl)	11.9 (9.1, 14.9)	13.1 (9.0, 15.0)
WBC (×10^9^/l)	12.0 (5.7, 17.7)	12.6 (9.9, 26.7)
CK-MB (IU/l)	23.9 (11.7, 71.7)	36.8 (17.5, 159.2)
Troponin I (pg/ml)	11 (00, 48)	13 (0, 772)
Blood sugar (mmol/l)	5.9 (4.2, 9.1)	5.7 (4.2, 10.9)
Lab-confirmed EV infection	25 (76)	12 (100)
EV-A71 positive	23 (70)	12 (100)
Other EV positive^f^	2 (6)	0

Summary statistic is absolute count (%) for categorical variables and median (range) for continuous data

^a^Features assessed within the 24 h before the actual/potential time to start MgSO_4_ (T = 0)

^b^Missing data for tachypnea in 4 cases in the MgSO_4_ group. For the lab investigations data were missing in less than 5% of cases for most variables, except that CK-MB and Troponin were only measured in patients with tachycardia sustained > 170 bpm (MgSO_4_ Group – 18 cases, Control group – 6 cases)

^c^Skin manifestations of autonomic nervous system (ANS) dysregulation

^d^Tachycardia: Heart rate sustained > 150 beats/min, adjusted down by 10 for each 1oC of fever above 37.0 °C

^e^Myoclonic jerks were not observed during the 24 h before T = 0 in any patient, while limb tremor/ataxia was present in 1 case in the MgSO_4_ exposed group

^f^Including 1 case with Coxsackievirus (CV) A 16, 1 case with an undefined enterovirus, all other cases PCR negative

Ten deaths occurred within the specified time period, nine in 2011 and one in 2012. Six patients developed severe hypertension and deteriorated very rapidly before reaching the maximum dose of milrinone, and none of the 10 patients fulfilled the selection criteria for this analysis. In the selected study population, no child died or progressed to shock, although three children, two controls and one MgSO_4_ recipient, required ventilation. There was no significant difference in the primary outcome between the MgSO_4_ and control groups (relative risk (95%CI) of 0.16 (0.01, 1.79), *p* = 0.132) (Table 2, Panel B). Similarly, we found no difference in the AUCs for SBP and MAP above the Stage 1 hypertension level between the groups (Table 3).

### Therapeutic monitoring

**Fig. 3:**
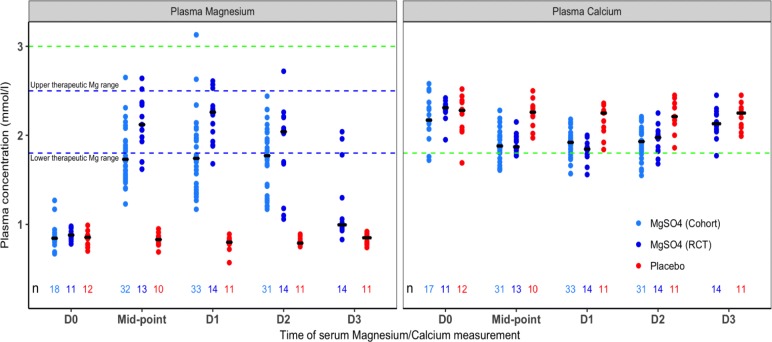
Combined data for the serial Magnesium/Calcium levels in the clinical trial and the observational cohort. Legends: In the observational cohort Mg/Ca levels were only measured in patients receiving MgSO_4_. Black bar = median value; n = number of patients assessed. Dashed green line: Potentially toxic levels for hypermagnesemia and hypocalcemia. D0 = baseline values, Mid-point = values measured 8–16 h after commencing the study drug. Other values (D1, D2, D3) were measured at 8–10 am on the relevant study day. By study day 3 a number of the trial patients had completed 72 h of the intervention and were already being weaned off their study drug. Patients in the observational cohort generally stopped MgSO_4_ after 48 h

In both patient groups that received MgSO_4_, plasma Mg levels increased within a few hours. Almost all trial participants achieved levels in the desired therapeutic range (1.8 to 2.5 mmol/l), within the first 12 h (Fig. 3). However, Mg levels were generally lower in the observational cohort; although the treatment regimen was nominally the same in both groups, the peak infusion rate (median, IQR) achieved for the trial participants was 50 (40, 50) mg/kg/hr. compared to 40 (30, 50) mg/kg/hr. in the observational cohort. A total of 7/130 (5%) Mg levels measured in the MgSO_4_ recipients in the trial were in the 2.5–3 mmol/l range, resulting in a recommendation by the independent safety monitoring doctor to reduce the dose in 6 cases. In the observational cohort, 2 Mg levels were documented in the 2.5–3 mmol/l range, plus a single high value of 3.13 mmol/l. However, this child had no respiratory problems, the ECG was normal, and the Mg level dropped promptly to the therapeutic range following dose reduction.

The median Ca levels in the MgSO_4_ group decreased from 2.28 mmol/l at baseline to a minimum of 1.85 mmol/l on Day 1 then increased again to 2.13 mmol/l on Day 3, demonstrating a reciprocal relationship with the Mg levels (Fig. 3). In total 32 Ca values, including 20 in the observational study MgSO_4_ group, 11 in the trial MgSO_4_ group and 1 in the placebo group, were documented below the level of 1.8 mmol/l (equivalent to an ionised Ca^2+^ level of 0.9 mmol/l, i.e. the threshold for potential concern), with the lowest recorded value of 1.55 mmol/l. No clinical AEs linked to low Ca levels occurred and no interventions were required.

## Discussion

Therapeutic options for severe HFMD remain limited [1, 3, 4], despite the rapidly increasing burden of disease in the southeast Asian region over the last two decades, and the life-threatening nature of the clinical syndromes seen in a small proportion of cases [33–37]. In this report we present the findings from two studies examining the efficacy and safety of intravenous MgSO_4_ for management of ANS related hypertension in children with HFMD complicated by brainstem encephalitis.

Regrettably, the first study, a formal randomized double-blind placebo-controlled clinical trial, had to be stopped on the grounds of futility after enrolment of only 26 of the planned 190 cases when the regional epidemic of HFMD waned after 2014. Baseline information was similar between the two treatment arms, except that a greater proportion of children in the placebo group presented with more severe (Stage 2) hypertension. Conversely, EV-A71, the serotype generally associated with more severe outcomes [38, 39], predominated in the MgSO_4_ group. These differences between the treatment arms are likely to be random and related to the small number of participants recruited [40], but there is the potential for an impact on the outcomes of interest.

With respect to efficacy, although we observed minor differences in the findings for a number of outcomes, generally favoring the MgSO_4_ group, there were no significant differences in any of the primary and secondary outcomes evaluated in the trial. However, given the small number of patients enrolled and the low event rate, no conclusions can be drawn. The initial pilot data had suggested an effect in patients with Stage 2 hypertension who were already on milrinone [21], and it is possible that among the less severe cases enrolled in the formal clinical trial, any potential beneficial effects were diluted.

More patients were included in the observational cohort analysis than in the clinical trial, but the final results also showed no statistically significant difference in the primary or secondary endpoints between those patients who did and did not receive MgSO_4_. However, the differences we noted between the two groups – i.e. older age and a lower proportion of participants with EV-71 infection in the exposed than the control group – are both typically associated with less severe disease outcomes and may be confounders. In addition, the patients included in the observational cohort were generally more severe at baseline than those enrolled in the clinical trial (Stage 2 versus Stage 1 hypertension), and the Mg levels were generally lower than the levels achieved in the trial. In studies of MgSO_4_ use in other conditions, the target therapeutic level has varied from 2 mmol/l to 5.5 mmol/l [10, 19, 41]. For the trial we selected a target range of 1.8–2.5 mmol/l, with most patients achieving the upper end of this range early on. By contrast, more than half the patients in the observational cohort who received MgSO_4_ did not achieve the 1.8 mmol/l lower margin, most likely because this was a new intervention in very sick children and clinicians were more cautious during the first year until they developed confidence that serious toxicity did not occur. Thus, it is possible that a therapeutic effect might have been demonstrated had we aimed for higher Mg levels.

Limited data are currently available regarding MgSO_4_ safety profiles in children [14, 19, 42], or the relationship between different therapeutic regimens and measured Mg and Ca levels. Our data are important in showing that potentially toxic Mg levels very rarely occurred using 30–50 mg/kg/hr. in sick children. Also, use of this regimen resulted in consistent plasma Mg levels with reciprocal plasma Ca responses in most study participants, indicating that detailed laboratory monitoring may not be necessary in the absence of clinical concerns suggesting toxicity. However, should more aggressive regimens be adopted in future studies, aiming to investigate efficacy at higher plasma Mg levels, regular monitoring of plasma levels would still be required.

## Conclusions

To summarize, in these two studies we found no clear evidence of benefit but a dose of 30-50 mg/kg/hr. MgSO_4_ was safe in these children with severe HFMD. However, it is apparent that these data are not sufficient to address the hypothesis originally posed. The question of whether MgSO_4_ could be optimized as first line therapy remains an important one, and an adequately powered trial is still needed to properly evaluate its role in controlling hypertension in severe HFMD. Epidemiological observations over the last 20–30 years suggest that ongoing outbreaks of HFMD are likely to occur intermittently across the region in the coming years. To facilitate clinical research during epidemics of infectious disease advance preparation is crucial. The experience gained from these studies, both the general experience of setting up and conducting a complex trial on sick children during a major outbreak, and the more particular information regarding the positive safety profile of MgSO_4_ used in this way plus the information generated on dosing in relation to plasma Mg/Ca levels, should prove invaluable when the next epidemic occurs.

## Additional files

Additional file 1: **Appendix A.** Details of the general study methodology for the clinical trial. **Appendix A.1.** Trial study_Screening and enrolment. **Appendix A.2.** Trial study_Sampling. **Appendix A.3.** Trial study_ Initiation of study medication, safety monitoring, dose adjustment. **Appendix A.4.** Trial study_Emergency management. **Appendix A.5.** Trial study_Emergency unblinding procedure. **Appendix A.6.** Trial study_Additional study definitions. **Appendix A.7.** Trial study_Definitions for Clinical Adverse Event Grading in the trial (modified from CTCAE Version 4.03). **Appendix A.8.** Trial study_Definitions for Laboratory Adverse Event Grading in the trial (modified from CTCAE Version 4.03). **Appendix B.** Additional methods for the observational cohort study. **Appendix B.1.** Cohort study_Identification of study subjects. **Appendix B.2.** Cohort study_Data collection and data management. **Appendix B.3.** Cohort study_Statistical analysis. (ZIP 257 kb)

Additional file 2: **Table S1.** Brief Summary of the Vietnamese MoH Classification for HFMD. (DOCX 81 kb)

Additional file 3: **Table S2.** Comparison of Bayley-III neurodevelopmental assessments 6 months after discharge for the clinical trial participants. (DOCX 78 kb)

Additional file 4: **Table S3.** AUCs of systolic blood pressure above the stage 1 hypertension cut-off, comparisons between groups who received MgSO_4_ and those who did not, for each imputed dataset plus the overall pooling. (DOCX 93 kb)

Additional file 5: **Table S4.** AUCs of mean arterial pressure (MAP) above the stage 1 hypertension cut-off, comparisons between groups who received MgSO_4_ and those who did not, for each imputed dataset plus the overall pooling. (DOCX 31 kb)

Additional file 6: **Figure S1.** The evolution of plasma and urine catecholamine levels over time in the two study arms. (DOCX 731 kb)

## Data Availability

The datasets used and/or analysed during the current study available from the corresponding author on reasonable request. Data was deposited at Harvard Dataverse. https://dataverse.harvard.edu/dataset.xhtml?persistentId=doi:10.7910/DVN/SP6HE7

## Abbreviations

AEs: Adverse events
ANS: Autonomic Nervous System;
AUC: Area Under The Curve
BP: Blood Pressure
CI: Confidence Interval
CNS: Central Nervous System
CTCAE: Common Terminology Criteria for Adverse Events
EV-A71: Enterovirus A71
HFMD: Hand Foot and Mouth Disease
HR: Heart Rate
HTD: Hospital For Tropical Diseases
IQR: Interquartile Range
MAP: Mean Arterial Pressure
MoH: Ministry Of Health
NO: Nitrite Oxide
PICU: Pediatric Intensive Care Unit
RT-PCR: Reverse Transcriptase Polymerase Chain Reaction
SAEs: Serious adverse events
SBP: Systolic Blood Pressure
