# Supplementary material for: Assessing the efficacy and safety of magnesium sulfate for management of autonomic nervous system dysregulation in Vietnamese children with severe hand foot and mouth disease
Source: BMC Infect Dis. 2019 Aug 22;19:737. doi: 10.1186/s12879-019-4356-x (PMC6704683; doi:10.1186/s12879-019-4356-x)
Supplement: Supplementary file 1 — Appendix A. Details of the general study methodology for the clinical trial. Appendix A.1. Trial study_Screening and enrolment. Appendix A.2. Trial study_Sampling. Appendix A.3. Trial study_ Initiation of study medication, safety monitoring, dose adjustment. Appendix A.4. Trial study_Emergency management. Appendix A.5. Trial study_Emergency unblinding procedure. Appendix A.6. Trial study_Additional study definitions. Appendix A.7. Trial study_Definitions for Clinical Adverse Event Grading in the trial (modified from CTCAE Version 4.03). Appendix A.8. Trial study_Definitions for Laboratory Adverse Event Grading in the trial (modified from CTCAE Version 4.03). Appendix B. Additional methods for the observational cohort study. Appendix B.1. Cohort study_Identification of study subjects. Appendix B.2. Cohort study_Data collection and data management. Appendix B.3. Cohort study_Statistical analysis. (ZIP 257 kb) [file 12879_2019_4356_MOESM1_ESM.zip › Appendix A.1 - Trial study_Screening and enrolmentR4.docx]

**Appendix A.1: Trial study_Screening and enrolment**

**Pre-screening**

- Identify potential study patients in PICU:-
  - Age 6 months to 15 years
  - Clinically diagnosed Grade 2b and 3 HFMD (see Table S1) with a medical indication for invasive blood pressure (IBP) monitoring
- Open the Height Percentile Calculator on the desktop ward computer. Enter the sex, age in months and height of the child to calculate the height percentile. Choose the appropriate *BP Label* from the study binder according to gender, age, and height percentile. Have a different person double-check all values and the chosen label. Stick the *BP Label* on the inside cover of the patient’s hospital file
- Provide a Screening Number using the next available number from the *Screening and Enrolment Log.* Complete the *Screening & Enrolment Log* as the steps below are being performed. Ensure that:-
  - Arterial line/catheter is in place
  - Backup venous line is available in addition to the main line
  - Appropriate IBP monitoring system has been established
  - Standard laboratory tests (WBC, CRP, Creatinine) are being performed
  - Blood sugar level is recorded in the hospital observation chart every 6 (+/-2) hourly
- Explain study information to the parent/guardian and give them the *Informed Consent Form* (ICF) to read
- Connect the Nihon Kohden/Space Labs monitor to the LiDCo monitor (Refer to the *Operating Monitors SOP)*
  - Study doctor should set the alert at the 95^th^ percentile (from *the BP Label*) of SBP on Nihon Kohden
  - Go to menu screen, choose PRESS button. Set the alert to Emergency Signal. The monitor will alert if SBP meets the set warning levels
  - Remember to calibrate the monitor before taking readings
- If the patient recovers and is discharged without developing hypertension as described below, complete the *Screening & Enrolment Log* up to the column on BP observations.

**Screening**

***If a patient develops hypertension (Grade 1 = SBP above 95th percentile, Grade 2 = SBP above 99th percentile + 5mm Hg), verify the INCLUSION CRITERIA below with the available observations:-***

- For children aged 1 year and over, at least 3 consecutive systolic blood pressure recordings above the 95^th^ centile for age, gender and length measured invasively over a period of 20 minutes provided the child is not distressed or crying. Note the three SPB readings on the hospital file.
- For children aged 6 months to 1 year, systolic BP > 100 mm Hg measured invasively on at least 3 occasions over a period for 20 minutes provided the child is not distressed or crying.

A paper record containing these readings should be printed out from the cardiac monitor and signed/dated by a study staff member, with the study number recorded on it. These records are to be included in the CRF, with a copy also included in the hospital file.

- **PLUS ONE OR MORE** of the following inclusion criteria:-
- Tachypnea for age
- Irregular or labored breathing, but with SpO_2_ above 92% in air and normal ABG
- Resting heart rate > 150 bpm
- Mottled skin
- Profuse sweating
- Refractory fever
- Hyperglycemia (>150 mg/dl on a random test, or >126 mg/dl on a fasting test (at least 4 hours after feeding)
- Verify the following **EXCLUSION CRITERIA** with available observations.
- Past history of hypertension, chronic renal, cardiac or pulmonary disease, or any neurological disorder
- Hypertensive emergency
- Already commenced milrinone or any other inotropic agents
- Respiratory distress with SpO_2_<92% in air or PaCO_2_>45 mm Hg
- AV block or any arrhythmia
- Acute renal failure: Creatinine> 2x upper normal range for age* or urine output <1ml/kg/hr for 4 hours (*UNR = >6<12 mth: 18 - 35 μ/L; >12 mth <12 yrs: 27 – 62 μ/L; >12 yrs: 44 – 88 μ/L)
- If the patient is excluded based on a permanent criterion complete a SCREENING CRF form and finalise the remaining information in the *Screening & Enrolment Log*. Stop all study related procedures.
- If the patient does not currently meet the inclusion/exclusion criteria due to a clinical feature that may change, continue to follow the patient and re-evaluate criteria regularly.
- If the patient meets all inclusion criteria and does not meet any exclusion criteria based on available information (you may not have an ABG or ECG yet) approach the parent/guardian for informed consent.

**Informed consent**

Study staff will discuss the study with the accompanying parent/guardian. If both parents are dead or not actively involved in caring for the child, the main long-term carer for the child will be accepted as a guardian and considered able to give consent for the study.

- Study staff will describe the purpose, procedures, risks/benefits, rights/responsibilities of participants, and alternatives to enrolment. The parent/guardian will be invited to ask questions which will be answered by study staff.
- If the parent/guardian agrees for the child to participate, they will be asked to sign and date an ICF. A copy of the form will be given to them to keep.
- If the parent/guardian is illiterate, the ICF will be read to them in the presence of a witness who will sign to confirm the parent/guardian’s understanding and acceptance.
- The study staff taking consent must also sign and date the ICF. The name of the parent/guardian and study staff (and witness if applicable) can be filled in by anyone.

**Refusal of consent** - If the parent/guardian refuses consent for any reason, complete the *Screening & Enrolment Log* and a SCREENING CRF.

**After consent is given**

- If an ABG was not done within the previous 2 hours (or was abnormal) take 0.5ml of blood from the arterial line and make sure the results are available as soon as possible (within 30 minutes). Check results against inclusion criteria above.
- If an ECG was not done within the previous 2 hours, perform a bedside ECG. Check results against the exclusion criteria. In very rare cases where it is not possible to perform a full ECG due to the irritability of the child and the need to commence intervention as soon as possible, the study doctor will observe the cardiac rhythm on the monitor and check against the exclusion criteria. A full ECG should then be performed as soon as possible after enrolment.
- If creatinine was not done within the previous 4 hours (or was abnormal) repeat it urgently and check against the inclusion criteria

**If the child is not eligible for the study -** Inform the parents/guardians of the reason for exclusion. Complete the SCREENING CRF and the *Screening and Enrolment Log* up to the “Enroll” column. Place the SCREENING CRF in the *Failed Screening* section of the study binder.

**Enrolment**

***Make sure that the maximum time from development of hypertension to commencing study treatment is <30 mins for patients with Grade 2 hypertension and <60 mins for patients with Grade 1 hypertension. These times should be written on the SCREENING CRF and the STUDY DRUG CRF.***

**If the child is enrolled to the study: –**

- Print out the data stored in the hemodynamic profile of the patient’s monitor for the 2 hours preceding enrolment for inclusion in the CRF.
- Take the next sequential treatment pack from the study drug storage cabinet. Write the study number from the drug package on the *Screening & Enrolment Log* and complete the rest of the log. Write the study number on the SCREENING CRF. Place the SCREENING CRF into a full patient file. Write the patient’s name and weight on the study drug package. Complete the *Delivery and Return Study Drug* form. Commence treatment as soon as possible – refer to *Medication SOP and Inpatient SOP*
- Take the T0 (baseline) samples before the first dose of study drug if possible. The clinical doctor may decide on the necessity of repeating these tests in the case of a very recently available result.
- Combined nasal/throat swab and rectal swab for PCR diagnostics
- Stamp all *HTD Lab Request Forms* with the **02EI** stamp. Refer to the *Sampling SOP.*
- Complete the *Patient Contact Sheet* in the patient file.
- Do a full clinical assessment as soon as possible and complete the DEMOGRAHIC, HISTORY and EXAMINATION CRFs.

**In view of the initial need for rapid consent, make sure to review the patient information sheet with the family of all enrolled participants at least once more when the patient is stable.**
