# Supplementary material for: Assessing the efficacy and safety of magnesium sulfate for management of autonomic nervous system dysregulation in Vietnamese children with severe hand foot and mouth disease
Source: BMC Infect Dis. 2019 Aug 22;19:737. doi: 10.1186/s12879-019-4356-x (PMC6704683; doi:10.1186/s12879-019-4356-x)
Supplement: Supplementary file 1 — Appendix A. Details of the general study methodology for the clinical trial. Appendix A.1. Trial study_Screening and enrolment. Appendix A.2. Trial study_Sampling. Appendix A.3. Trial study_ Initiation of study medication, safety monitoring, dose adjustment. Appendix A.4. Trial study_Emergency management. Appendix A.5. Trial study_Emergency unblinding procedure. Appendix A.6. Trial study_Additional study definitions. Appendix A.7. Trial study_Definitions for Clinical Adverse Event Grading in the trial (modified from CTCAE Version 4.03). Appendix A.8. Trial study_Definitions for Laboratory Adverse Event Grading in the trial (modified from CTCAE Version 4.03). Appendix B. Additional methods for the observational cohort study. Appendix B.1. Cohort study_Identification of study subjects. Appendix B.2. Cohort study_Data collection and data management. Appendix B.3. Cohort study_Statistical analysis. (ZIP 257 kb) [file 12879_2019_4356_MOESM1_ESM.zip › Appendix A.2 - Trial study_SamplingR4.docx]

**Appendix A.2: Trial study_Sampling**

**Laboratory equipment kit preparation**

| Tubes | D1 | | | D2 | D3 | D4 | Discharge |
| --- | --- | --- | --- | --- | --- | --- | --- |
|  | Screening | T0 | T12 | T24 | T48 | T72 |  |
| EDTA | 1 | 2 | 1 | 2 | 1 | 1 | 1 |
| Li-Heparin | 1 |  | 1 | 1 | 1 | 1 |  |
| Urine collecting bag |  | 1 |  | 1 | 1 | 1 |  |
| VTM tube |  | 2 |  |  |  |  |  |
| Preservatives container 1.5 litre |  | 1 |  | 1 | 1 | 1 |  |
| Urine-Aliquot 2ml |  |  |  | 2 | 2 | 2 |  |

Ensure all necessary blood tubes are prepared in advance in accordance with the table above and stored in zip lock bags with appropriate labels assigned for each study day. These packages are kept in the study cabinet on the ward, except that containers with Viral Transport Medium for the diagnostic swabs will be stored in the ward fridge. The labels for each sample should be kept in the study binder. For each participant five 1.8 liter urine collecting bottles containing 10 ml of 6M HCL should be prepared in the OUCRU Lab (as below) and send to PICU in advance.

- - Add 10 ml aliquots of 6M HCL solution to multiple 1.5 liter urine bottles and store at the OUCRU laboratory
  - Check the pH every month to make sure the preservative solution is stable. If the pH changes, replace new solution in the prepared bottles.
  - Study nurse will ensure there are always five bottles available at PICU for a potential new study participant

**Blood sampling**

To fit in with routine ward practice, all samples except those obtained at enrolment will be taken at 8am or 8pm. All patients should have blood taken approximately 12 and 24 hours after enrolment, scheduled to coincide with the routine 8am and 8 pm venesection rounds. After this, for the next 2 days, all patients will have an 8 am blood draw, which will be considered as the 48 and 72 hr draws.

Ensure every time a sample is taken the following steps are done:-

- - A label with the appropriate randomization number and the type of specimen must be stuck on every sample tube. Also write on the label the date and time the sample is taken, and fill in the *Sample Tracking Form*. For hospital samples also complete the relevant *Hospital Request Form.*
  - The study nurse must make sure all the necessary tests on the checklist are done and ticked when performed. If not done, remind the doctors immediately to give orders.
- **During screening (**ABG and Blood sugar**)**
- 0.5 ml arterial blood will be drawn from the indwelling line for arterial blood gas (ABG), for patients whose parent/guardian has given consent to enroll in the trial.
- After using a drop of blood to perform a blood sugar measurement using the standard ward equipment, inject the remaining blood to the Li-Heparin tube
- Fill the “02EI screening number” on the *Label* and paste to the tube
- Send it directly to Biochemistry Lab as soon as possible with **02EI** stamped on the *Hospital Request Form*, (transported by a study nurse or medical assistant)
- The lab staff will receive the sample and perform ABG as soon as possible
- The result will be uploaded directly to the Hospital’s network system
- The study doctor will check the result in the PICU computer. In case the study staff don’t receive the result after 15 minutes, she/he will call to Lab, give the screening number and patient name and obtain the result
- If the hospital network system fails, the medical assistants will be waiting at the lab to collect the result (approximately 10-15 minutes). The results will be informed to the study doctors at PICU ward via the 02EI study phone, or by direct contact with the study doctor’s mobile.
- If an ABG was performed within 2 hrs (and is normal), this result can be used for screening
- **At T0 (enrolment)**
- Hospital Lab: 1.5-2ml Li-Heparin for Biochemistry (Electrolytes-including Mg and Ca, Creatinine, CKMB, Troponin, Glycemia).
- The request for Ca & Mg tests must be separated from the other forms. On the request form for Ca &Mg, indicate clearly that the lab staff should contact the Mg Safety Monitoring Doctor (MSMD) on duty, according to the rota displayed in the lab. The form should also be clearly stamped with the following message in red – “DO NOT UPLOAD THIS RESULT TO THE COMPUTER SYSTEM” and “DO NOT RETURN THIS RESULT TO PICU”
- OUCRU Lab: 2 ml EDTA for catecholamines, 1 ml EDTA for serology and cytokine.

If FBC and CRP have not been done within the previous 24 hours, make sure these tests are done also.

- **At T12 (around 12 hours after enrolment)**

If the T12 blood draw falls due after less than 6 hours from the enrolment time, it should be delayed to the next nearest 8am/8pm draw. Take the following samples from the arterial line

- For the hospital lab: 1 ml Li-Heparin for Biochemistry (Mg, Ca).
- For the OUCRU lab: 1 ml EDTA.
- A drop of blood from the syringe should be used to perform blood sugar measurement using the standard ward equipment.
- The request for Ca & Mg test must be separated from the other forms, following the instructions as for T0.
- **At T24 (around 24 hours after enrolment)**
- For OUCRU: 2 ml EDTA for catecholamines, and 1 ml for cytokine
- For hospital routine: 1.5-2ml Li-Heparin for Biochemistry (Electrolytes-including Mg and Ca, Creatinine, CKMB, Troponin, Glycemia), plus a ward blood sugar measurement
- The request for Ca & Mg test must be separated from the other forms, following the instructions as for T0.
- **At T48, T72**
- For OUCRU: 2 ml EDTA for catecholamines (at T48 ONLY)
- For hospital routine: 1.5-2ml Li-Heparin for Biochemistry (Electrolytes-including Mg and Ca, Creatinine, CKMB, Troponin, Glycemia), plus a ward blood sugar measurement
- The request for Ca & Mg test must be separated from the other forms, following the instructions as for T0.

**At T96**

- For OUCRU: 2 ml EDTA for catecholamines

**Urine sampling**

All urine should be collected into the prepared 1.5 liter bottles. Urine aliquots for lab studies should be taken at 6 am each day from these bottles (after shaking well) and sent to OUCRU Lab at 8 am everyday.

- At enrolment, take one 1.5 liter bottle, complete the label (study number, date, time) and put at the bedside
- Attach the urine collecting bag to the patient (Urinocol- B/Braun)
- Make sure the study nurse empties the urine from the collecting bag regularly, at least every 4 hours
- Empty the urine bag to the sample urinal jug and measure the volume, then pour the urine into the collecting bottle and shake
- Record the total volume at the end of the 24 hour period on the *Nursing Chart*, and place a fresh 1.5 liter bottle at the bedside
- Each morning, take 2 aliquots of urine (1.5 ml each) form the 1.5 liter bottle, fill in the OUCRU label for the samples and the *Sample Tracking Form* and then send to the OUCRU lab, for storage at -70^o^C

**Diagnostic specimens**

A nasal/throat swab and a rectal swab should be taken at enrolment for PCR diagnostics.

- If taken before 4 pm
- Fill in the OUCRU label, including date/time, for the sample
  - 02EI Study nurse or PICU medical assistant should deliver to OUCRU virology lab for processing
  - Record the sample information into the *Specimen Tracking Log*.
- If taken after 4 pm
- Fill in the OUCRU label, including date/time for the sample
- Store the samples in the PICU fridge at 4 degrees
- Record the sample information into the “*Specimen Tracking Log*.
- The study nurse will deliver the samples to the virology lab at 8 am the next morning for processing

**OVERALL SAMPLING SCHEDULE**

|  | | D1 | | D2 | D3 | D4 | D5 | Disc |
| --- | --- | --- | --- | --- | --- | --- | --- | --- |
| Hour | | T0 | T12 | T24 | T48 | T72 | T96 |  |
| Biochemistry  (Li-Heparin) | Creat, Na, K, Cl, Ca&Mg TnI, CK-MB, Glucose: 1.5 ml | ✓ |  | ✓ | ✓ | ✓ |  |  |
|  | Mg/Ca only: 1ml |  | ✓ |  |  |  |  |  |
|  | ABG (heparin): 0.5ml | ✓ |  | ✓ | ✓ | ✓ |  |  |
| OUCRU  3ml (EDTA) | 2ml for catecholamine | ✓ |  | ✓ | ✓ |  | ✓ |  |
|  | 1ml for cytokine | ✓ | ✓ | ✓ |  |  |  | ✓ |
| Serology | | ✓ |  |  |  |  |  | ✓ |
| Urine 5ml | |  |  | ✓ | ✓ | ✓ |  |  |
| Diagnostic swabs | | ✓ |  |  |  |  |  |  |
| Amount of blood needed (ml) | | 5 ml | 2 ml | 5 ml | 4ml | 2ml | 2 ml | 2ml |
| Total (day) | | 7 ml | | 5 ml | 4 ml | 2 ml | 2ml | 2ml |
