# Supplementary material for: Assessing the efficacy and safety of magnesium sulfate for management of autonomic nervous system dysregulation in Vietnamese children with severe hand foot and mouth disease
Source: BMC Infect Dis. 2019 Aug 22;19:737. doi: 10.1186/s12879-019-4356-x (PMC6704683; doi:10.1186/s12879-019-4356-x)
Supplement: Supplementary file 1 — Appendix A. Details of the general study methodology for the clinical trial. Appendix A.1. Trial study_Screening and enrolment. Appendix A.2. Trial study_Sampling. Appendix A.3. Trial study_ Initiation of study medication, safety monitoring, dose adjustment. Appendix A.4. Trial study_Emergency management. Appendix A.5. Trial study_Emergency unblinding procedure. Appendix A.6. Trial study_Additional study definitions. Appendix A.7. Trial study_Definitions for Clinical Adverse Event Grading in the trial (modified from CTCAE Version 4.03). Appendix A.8. Trial study_Definitions for Laboratory Adverse Event Grading in the trial (modified from CTCAE Version 4.03). Appendix B. Additional methods for the observational cohort study. Appendix B.1. Cohort study_Identification of study subjects. Appendix B.2. Cohort study_Data collection and data management. Appendix B.3. Cohort study_Statistical analysis. (ZIP 257 kb) [file 12879_2019_4356_MOESM1_ESM.zip › Appendix A.3 - Trial_Study medication_safety monitoringR4.docx]

**Appendix A.3: Trial study_ Study medication, safety monitoring, dose adjustment**

**Initiation of study medication**

- The study doctor must set the alert thresholds for SBP on the cardiac monitor
- Go to menu screen, choose PRESS button, set up the upper and lower range of SBP based on the BP label that is attached in the hospital file
- A> If the patients is enrolled at hypertension stage 1 (>95^th^ percentile, <99^th^ % +5), the upper alarm should be set at 99^th^ percentile + 5 mmHg, while the lower alarm will be set at 70 + 2*Age.
- B> If the patients is enrolled at hypertension stage 2 (>99^th^ + 5 mmHg), the upper alarm will be set at 99^th^ percentile + 15 mmHg, while the lower alarm will be set at 70 + 2*Age.
- Set the alert to Emergency Signal. The monitor will alert if SBP meets the set warning levels
- Study doctor should prescribe the study treatment in the hospital file:
- Study drug: 45 ml
- 0.5 ml x weight (kg) infused over 20 minutes
- Then infuse with 0.3 ml x weight (kg)/hr continuously
- This dose will be adjusted according to the clinical scenarios below and all changes should be noted in the hospital file as they occur
- The study nurse prepares the study drug as follows:
- Mix 3 study drug vials (10 ml) and 15 ml NaCl 0.9% in a 50 ml syringe to obtain 45 ml of final preparation
- Calculate the Loading Dose according to the patient’s weight

| **Study drug** | **Volume** | **Infusion time** | **Rate (ml/hr)** |
| --- | --- | --- | --- |
| Loading dose | 0,5 ml x weight (kg) | 20 minutes | 0,5 x weight x 3 |

- Set the rate of the infusion of the pump.
- Start the infusion and set the pump alarm to alert after 20 minutes.
- Calculate the Maintenance Dose according to the patient’s weight

| **Study drug** | **Rate (ml/hr)** | **Infusion time** |
| --- | --- | --- |
| Maintenance dose (continuous) | 0.3-0.5 ml x weight (kg) | Up to 72 hours |

- The study drug is then constantly infused, following the doctor’s orders, progressively increasing the dose every 20 minutes until the 50 mg/kg/hr dose is reached. Subsequently this dose will be adjusted according to the Mg levels (directed by the independent MSMD) and the clinical situation. The study nurse will write down in the hospital file and record any new dose on the *Nursing Chart*.
- The study staff will monitor closely the volume of drug in the infusion syringe, and prepare a new dose in advance when the remaining volume in the syringe is around 2 ml.
- If a study drug vial is accidently broken, the study nurse will prepare a new solution and note on the Drug Delivery and Return Form the number of broken vials.

**Independent MgSO_4_ Safety Monitoring**

A group of independent experienced clinicians who are not involved with patient care on PICU have been trained to monitor the magnesium (Mg) and calcium (Ca) levels in the study participants. The main responsibilities of these Mg Safety Monitoring Doctors (MSMDs) are to record and closely monitor the Mg/Ca values of the randomized patients and to indicate to the ward staff if there is a need to adjust the study drug infusion rate, in accordance with specific written guidelines.

A schedule of assigned MSMDs (one main MSMD, and one substitute) is posted in the ward. The study nurse will maintain the schedule and arrange with the MSMDs to ensure that each MSMD is available for their allotted times and that the responsibilities transfer smoothly. The assigned MSMD will rotate each week on Monday. If the lab/PICU staff cannot contact the assigned MSMD within 15 minutes, they will contact the substitute according to the phone numbers provided each week.

**System set up**

A separate, controlled-access database will be established by the OUCRU IT staff, containing the randomized allocation for each patient as well as a list of randomly assigned sham dose adjustments for patients who are randomized to receive placebo. None of the ward or study staff have access to this database

Each assigned MSMD will be provided with a username and password to access to this database. Access is possible on any device with internet access, so that the MSMD need not be on the hospital site all the time.

When a new patient is enrolled, the study nurse will inform the duty MSMD of the study number and patient name, and upon receiving the results for the enrolment blood sample, the MSMD will enter and save these values into the corresponding study participant file.

**On the ward**

Mg/Ca levels will be checked upon enrolment, at 8 am or 8 pm after around 12 hours, and at 8 am on the next 2 sequential days.

Mg/Ca levels may also be checked at any point in the case of an emergency.

Mg/Ca tests will be requested and reported separately from other Biochemistry tests. Study staff will verify the schedule to know which MSMD is on duty that day and will apply a label with the name and phone number of the assigned MSMD on the request form for Mg and Ca test. The label will also include the instruction “Do not upload this result”.

**In the laboratory**

Mg/Ca tests with study labels will be processed as soon as possible. The lab staff will text and then call the number on the label to inform the duty MSMD of the results of the Mg/Ca levels, with the correct study number of the participant. Lab staff will make sure the Mg/Ca results are not uploaded onto the hospital computer system.

For the scheduled tests:

- If the Ca < 0.9 mmol/l the lab technician will inform the MSMD immediately. The MSMD will call the study/ward doctor immediately to inform them of the result.
- If the Mg > 3.0 mmol/l the lab technician will inform the MSMD immediately. The MSMD will call the study/ward doctor immediately to decrease the dose.
- For any other Ca/Mg results, the lab technician will inform the MSMD no later than 10 am daily for 8 am draws and no later than 10 pm for 8 pm draws (note that this will only occur at 8 pm on days when new patients are enrolled).

**MSMD Duties**

- When a result is received from the lab, the MSMD will repeat the result, time point and patient number back to the lab to double check the results. The MSMD will then enter the result for the appropriate patient number and time point in the controlled-access database and enter their recommended dose adjustment (for active Mg arm only – see decision criteria below)*.*
- After receiving the scheduled blood result, the MSMD will ring the ward and inform the staff of their recommendation no later than 11 am daily or 11 pm for evening draws.
- When enrolled patients are within the first 48 hours of treatment, if the MSMD does not receive a call from the lab by 10 am/pm, s/he will call the lab to check what happened
- On day 4 (around T72), before giving the recommendation the MSMD will ask the study doctor/treating doctor if the study patient is still on study drug. If the study patient has already completed the study drug course, the MSMD will complete the patient record and close that file.

**For patients on the placebo arm:**

The MSMD will call the study doctor and recommend that the dose be decreased, maintained or increased according to the randomly allocated sham adjustments indicated in the database, unless the current dose is already the maximum (if an increase is indicated) or minimum (if a decrease is indicated). In these cases, the recommendation should be No Change.

**For patients on the active MgSO_4_ arm:**

- If serum Mg <1.8 mmol/l - tell study doctor to increase dose by one increment
- If serum Mg >2.5 mmol/l – tell study doctor to decrease dose by one increment
- If serum Mg 1.8 - 2.5 mmol/l – tell study doctor not to change the dose

**In emergency circumstances**:

If knowing the Mg/Ca test result could be relevant to patient care, the study/treating doctor will discuss the emergency circumstance with the site PI immediately, and request an emergency Mg/Ca level. If the study doctor/treating doctor has any concerns, the result will be released immediately to the ward and the lab technician will inform the MSMD, then the MSMD will complete the *Mg Safety Monitor Log.* Otherwise, the MSMD will follow the instructions as above.

**Dose adjustment**

Study nurses will monitor the blood pressure each hour, during all study drug dose changes, and additionally according to clinical need. The study doctor will be available at the bedside to decide dose adjustments according to the following criteria, sometimes together with advice provided by the MSMD who has access to the Mg/Ca blood results (as indicated in the guidelines above):

|  |  | Study drug dose adjustment | |
| --- | --- | --- | --- |
| SBP status | Value | Without MSMD advice | MSMD advice – suggested action based only on blood levels is shown in bold italics (see Appendix A.6) |
| If SBP is decreasing | > 90th and ≤95th percentiles | No Change | ***Increase:*** adhere to advice if not on maximum dose (0.5.ml/kg)  ***Decrease:*** adhere to advice  ***No change:*** adhere to advice |
|  | ≤ 90th percentile and ≥ 70th percentile +2n | Decrease by 0.1 ml/kg every 15 minutes | ***Increase:*** do not follow  ***Decrease:*** adhere to advice if not at the minimum dose (0.1 ml/kg)  ***No change***: do not follow |
|  | Rapidly, more than 25% over 15 minutes | Decrease by 0.1 ml/kg every 15 minutes | ***Increase:*** do not follow  ***Decrease:*** adhere to advice  ***No change:*** do not follow |
|  | But fails to decrease by 25% over the first 8 hours despite achieving maximum dose of study drug | ADD MILRINONE | ***Increase:*** do not follow  ***Decrease:*** adhere to advice  ***No change***: adhere to advice |
| If SBP is stable or increasing slowly | > 95th percentile and ≤ 99th centile +5 and dose is less than 0.5 ml/kg/hr) | Increase by 0.1 ml/kg every 15 minutes if dose is less than 0.5 ml/kg/hr | ***Increase:*** adhere to advice if not on maximum dose (0.5.ml/kg)  ***Decrease:*** adhere to advice  ***No change:*** do not follow unless on maximum dose already |
| If SBP is increasing significantly | Between 99th centile +5-15 mmHg within 30 mins | Increase by 0.1 ml/kg every 15 minutes if dose is less than 0.5 ml/kg/hr | ***Increase:*** adhere to advice if not on maximum dose (0.5.ml/kg)  ***Decrease:*** adhere to advice  ***No change***: do not follow unless on maximum dose already |
|  | Between 99th centile +5-15 mmHg for > 30 mins | Increase by 0.1 ml/kg every 15 minutes if dose is less than 0.5 ml/kg/hr  AND ADD MILRINONE | ***Increase:*** adhere to advice if not on maximum dose (0.5.ml/kg)  ***Decrease:*** adhere to advice  ***No change***: do not follow unless on maximum dose already |
|  | SBP > 99th centile +15 mmHg within 15 mins | Increase by 0.1 ml/kg every 15 minutes if dose is less than 0.5 ml/kg/hr | ***Increase:*** adhere to advice if not on maximum dose (0.5.ml/kg)  ***Decrease:*** adhere to advice  ***No change:*** do not follow unless on maximum dose already |
|  | SBP > 99th centile +15 mmHg for >15 mins | Increase by 0.1 ml/kg every 15 minutes if dose is less than 0.5 ml/kg/hr  AND ADD MILRINONE | ***Increase:*** adhere to advice if not on maximum dose (0.5.ml/kg)  ***Decrease:*** adhere to advice  ***No change***: do not follow unless on maximum dose already |
| Hypertensive emergency: If SBP is increasing rapidly and there is evidence of acute target/end organ damage | SBP > baseline + 40 mmHg | Increase by 0.1 ml/kg every 15 minutes if dose is less than 0.5 ml/kg/hr  AND ADD MILRINONE | ***Increase:*** adhere to advice if not on maximum dose (0.5.ml/kg)  ***Decrease:*** adhere to advice  ***No change:*** do not follow unless on maximum dose already |

- **If patients are on both the study drug and milrinone when it is necessary to adjust the dose, please take note as follows**:
- ***Increasing blood pressure***: the dose of milrinone should be increased until the maximum dose (0,75 μg/kg/minute) is reached. If the SBP is still not controlled, please inform and discuss with PI to consider unblinding the study drug.
- ***Decreasing blood pressure***: the dose of milrinone should be reduced first until the minimum dose (0,4 μg/kg/ minute) is reached, followed by decreasing the study drug dose
- **If a patient is being treated with the maximum dose of milrirone as well as the maximum dose of study Mg/Placebo and the blood pressure is still not controlled:** the study doctor may consider unblinding the study medication to ensure the patient is receiving magnesium sulfate. Please discuss all decisions to unblind with the PI, and follow the *Unblinding Procedure* below.

**Please note also:**

- **Hypertensive emergency:** - A severe symptomatic elevation in BP (> 30% compared to baseline blood pressure) WITH evidence of acute target organ damage defines a hypertensive emergency
- Brain (seizures, increased intracranial pressure, paralysis)
- Kidneys (renal insufficiency)
- Eyes (papilledema, retinal hemorrhages, exudates)
- Heart (heart failure)
- **Stopping the study drug after 72 hours: -** Patients who complete the 72 hour study drug treatment at a dose of:
- 0.3 ml/kg/hr or less may stop study drug if their clinical condition permits
- >0.3 <0.5 ml/kg/hr should have the study drug tapered to 0.3 ml/kg/hr then stopped. Tapering should be done according to the patient's clinical condition and be completed within 6 hours when possible.

*Note: In rare cases, if the study doctor is not available at the bedside when a clinical decision is needed, the study nurse should contact the study doctor by phone to discuss the patient parameters. The study doctor will make a decision based on the pulse, RR, BP, Sp02 and other required clinical information. If the doctor decides to change the medication, the nurse will note this in the nursing record and the doctor will note this in the hospital file when s/he returns to the ward.*
