# Supplementary material for: Assessing the efficacy and safety of magnesium sulfate for management of autonomic nervous system dysregulation in Vietnamese children with severe hand foot and mouth disease
Source: BMC Infect Dis. 2019 Aug 22;19:737. doi: 10.1186/s12879-019-4356-x (PMC6704683; doi:10.1186/s12879-019-4356-x)
Supplement: Supplementary file 1 — Appendix A. Details of the general study methodology for the clinical trial. Appendix A.1. Trial study_Screening and enrolment. Appendix A.2. Trial study_Sampling. Appendix A.3. Trial study_ Initiation of study medication, safety monitoring, dose adjustment. Appendix A.4. Trial study_Emergency management. Appendix A.5. Trial study_Emergency unblinding procedure. Appendix A.6. Trial study_Additional study definitions. Appendix A.7. Trial study_Definitions for Clinical Adverse Event Grading in the trial (modified from CTCAE Version 4.03). Appendix A.8. Trial study_Definitions for Laboratory Adverse Event Grading in the trial (modified from CTCAE Version 4.03). Appendix B. Additional methods for the observational cohort study. Appendix B.1. Cohort study_Identification of study subjects. Appendix B.2. Cohort study_Data collection and data management. Appendix B.3. Cohort study_Statistical analysis. (ZIP 257 kb) [file 12879_2019_4356_MOESM1_ESM.zip › Appendix A.4 - Trial study_Emergency managementR4.docx]

**Appendix A. 4: Trial study_Emergency management**

If an emergency situation occurs (see examples described below), the study drug will be sopped immediately, the unblinding procedures must be followed and rescue management should be commenced:

1. Arrhythmia – AV block, QT >0.48ms, cardiac arrest
2. Urine output < 1ml/kg/hr > 4 hrs with evidence of acute renal failure
3. SBP < 70 mmHg
4. Respiratory complication requiring intubation / ventilation

**1.1 Prolong QT interval or any cardiac arrhythmia**

When plasma Mg level is high, usually above 5 mmol/l, this may result in prolongation of the QT interval (> 0.48 ms) and this could precipitate an arrhythmia. An ECG will be done at baseline and then daily and the QT interval will be measured as a routine. In addition if any arrhythmia is noted on the cardiac monitor the following actions will be taken.

- Assess the patient clinically, perform a full ECG, check plasma Mg and Ca level and discuss the clinical situation with the site PI.
- If the QT interval is above 0.48 ms, the study drug will be stopped immediately.
- Any other serious cardiac arrhythmia will be managed according to APLS guidelines
- The results of the Mg/Ca levels will go to the independent doctor for review. He/she will discuss the situation with the treating doctor. If the clinician has any clinical concerns the independent doctor will release the blood results so that the treating doctor can take any necessary action immediately. However, if the treating doctor and the site PI consider that the arrhythmia is minor (e.g. occasional SVE’s) and there are no other clinical issues of concern, then the blood results will not be released. The independent doctor may recommend adjustments to the study drug infusion according to the blood test results, but sham adjustments will also be made to the placebo arm to maintain blinding.
- However, if the calcium level < 0.9 mmol/l, the independent doctor will inform the treating doctor of this result specifically so that a rescue dose of 0.5ml/kg of calcium gluconate 10% can be given.

**1.2 Cardiac arrest**

In the event of a cardiac arrest the following actions will be taken:

- Stop study drug infusion immediately
- Emergency resuscitation will be started immediately according to APLS guidelines
- Plasma Mg and Ca levels will be checked urgently and the results will be returned directly to the ward clinicians.
- A rescue dose of 0.5ml/kg of calcium gluconate 10% will be given while awaiting the results if other attempts including CPR and conventional resuscitation drugs fail to restore sinus rhythm and effective cardiac output.
- The plasma Mg/Ca results may unblind the clinical team to the randomization arm and the fact that an urgent level has been reported to the ward doctors will be recorded in the CRF.
- Further intensive intervention will be done according to the Vietnamese MOH guidelines for HFMD management and APLS resuscitation guidelines.

**2. Urine output < 1ml/kg/hr**

If the urine output is < 1ml/kg/ hr over 4 hours the following actions will be taken:

- A bedside ultrasound will be performed to check whether there is any urine in the bladder
- A urethral catheter will be inserted to monitor the urine output closely, and the plasma creatinine will be checked urgently
- If the creatinine has increased to twice the baseline value, the study drug will be stopped and hemofiltration/dialysis will be performed according to the MOH guidelines regardless of the plasma Mg level.
- If the creatinine is in the normal range but the bladder is empty, then the urine output will be monitored closely for 4 hours more. If the urine output is increasing during this time the study drug will be continued, but if there is no improvement the study drug will be stopped and hemofiltration/dialysis will be performed according to the MOH guidelines regardless of the plasma Mg level.
- If the creatinine is in the normal range and there is urine in bladder, the urine output will be monitoring hourly for at least the next 24 hours

**3. Hypotension**

Development of hypotension could be the natural progression of severe disease or a side effect of MgS0_4_, especially if the plasma Mg level is above 3 mmol/l. If hypotension occurs, defined as a drop in systolic blood pressure to below 70mmHg + 2 X the age in years lasting for 15 minutes, the following actions will be taken:

- Study drug will be stopped immediately
- Assess the patient clinically, check plasma Mg/Ca levels and creatinine, and discuss the clinical situation with the site PI
- Start with a fluid challenge of 5ml/kg of Lactate Ringers or NaCl 0.9% over 15 mins
- Access the central venous pressure (CVP) and titrate the rate of fluid infusion based on CVP measurements and the clinical response.
- Start inotropes or vasopressors (such as dobutamine, noradrenaline or adrenaline), and continue with interventions following the Vietnamese MOH guideline.
- The results of the Mg/Ca levels will go directly to the ward clinicians. If the calcium level is < 0.9 mmol/l, regardless of the plasma Mg level, a rescue dose of 0.5ml/kg of calcium gluconate 10% will be given.
- The plasma Mg/Ca results may unblind the clinical team to the randomization arm and the fact that an urgent level has been reported to the ward doctors will be recorded in the CRF.
- If the creatinine level is increasing and the treating doctor suspects a diagnosis of acute renal failure, there may be a risk of toxicity due to Mg accumulation. Hemofiltration will be commenced according to MOH guidelines regardless of the plasma Mg level.

**4. Respiratory muscle weakness**

If the patient develops new or worsening signs and symptoms of respiratory distress the following actions will be taken:

- Assess the patient clinically including deep tendon reflexes, check the plasma Mg and Ca levels and an ABG and discuss the clinical situation with the site PI.
- If the patient meets the MOH ventilation criteria, (or the treating doctor thinks that the patient needs to be intubated for any reason) the study drug will be stopped immediately,
  - The results of the Mg/Ca levels will go directly to the ward clinicians. If the calcium level is < 0.9 mmol/l, regardless of the plasma Mg level, a rescue dose of 0.5ml/kg of calcium gluconate 10% will be given.
  - The plasma Mg/Ca results may unblind the clinical team to the randomization arm and the fact that an urgent level has been reported to the ward doctors will be recorded in the CRF.
- If the patient does not meet the MOH ventilation criteria and the treating doctor and site PI agree that immediate intervention/respiratory support is not needed the patient will be observed closely for at least 60 minutes.
  - The results of the Mg/Ca levels will go to the independent doctor for review as soon as possible. If the Mg level is above 2.5 mmol/l but below 3 mmol/l, the treating doctor will be informed to reduce the infusion dose as described in the protocol. Similar sham adjustments can also be made to the placebo arm to maintain blinding.
  - If the Mg level is 3 mmol/l or above, the independent doctor will release the result and inform the study doctor to stop the study drug infusion.
  - If the Ca level is < 0.9 mmol/l the treating doctor will be informed so that a rescue dose of 0.5ml/kg of calcium gluconate 10% can be given if appropriate.
  - If the patient subsequently meets the criteria for intubation, or the PaCO_2_ rises >45 mmHg, the intubation steps described above will be acted upon.

Any further interventions will be performed according to the Vietnamese MOH guidelines for HFMD management
