# Supplementary material for: Assessing the efficacy and safety of magnesium sulfate for management of autonomic nervous system dysregulation in Vietnamese children with severe hand foot and mouth disease
Source: BMC Infect Dis. 2019 Aug 22;19:737. doi: 10.1186/s12879-019-4356-x (PMC6704683; doi:10.1186/s12879-019-4356-x)
Supplement: Supplementary file 1 — Appendix A. Details of the general study methodology for the clinical trial. Appendix A.1. Trial study_Screening and enrolment. Appendix A.2. Trial study_Sampling. Appendix A.3. Trial study_ Initiation of study medication, safety monitoring, dose adjustment. Appendix A.4. Trial study_Emergency management. Appendix A.5. Trial study_Emergency unblinding procedure. Appendix A.6. Trial study_Additional study definitions. Appendix A.7. Trial study_Definitions for Clinical Adverse Event Grading in the trial (modified from CTCAE Version 4.03). Appendix A.8. Trial study_Definitions for Laboratory Adverse Event Grading in the trial (modified from CTCAE Version 4.03). Appendix B. Additional methods for the observational cohort study. Appendix B.1. Cohort study_Identification of study subjects. Appendix B.2. Cohort study_Data collection and data management. Appendix B.3. Cohort study_Statistical analysis. (ZIP 257 kb) [file 12879_2019_4356_MOESM1_ESM.zip › Appendix A.5 - Trial study_Emergency unblinding procedureR4.docx]

**Appendix A.5: Trial study_Emergency unblinding procedure**

When considering unblinding due to an Adverse Event, the study/treating clinician should contact the site PI, to discuss whether the following criteria are met:

1. The Adverse Event must be a Serious Adverse Event as defined in the protocol
2. The Serious Adverse Event must be thought to be probably or definitely related to the study drug as defined in the protocol
3. The treating clinician states that knowledge of the treatment arm may change the therapy provided to the participant or potentially improve the patient’s outcome.

**Unblinding procedure**

- The contact person will verify that the participant has met the criteria for unblinding and provide the unblinding information to one of the site PIs.
- One of the PIs must confirm that a serious adverse event has occurred.
- The site PI will review the above criteria for emergency unblinding and confirm that knowing the treatment arm of the study will aid or change the clinical management of the participant.
- The PI will complete the SAE Report Form and submit to the Data and Safety Monitoring Committee, to the manufacturers of the therapy (if the patient is randomized to the Magnesium arm of the study) and to Dr. Nguyen Van Vinh Chau, Director of HTD or Dr Nguyen Thanh Hung, Director of CH1, within 24 hours of becoming aware of the event, then send reports to MoH as the above procedure.
- The PI will call the study pharmacist to request the unblinding information. The PIs will be responsible for ensuring that the unblinding is recorded in the study file by the study nurse.
