# Supplementary material for: Assessing the efficacy and safety of magnesium sulfate for management of autonomic nervous system dysregulation in Vietnamese children with severe hand foot and mouth disease
Source: BMC Infect Dis. 2019 Aug 22;19:737. doi: 10.1186/s12879-019-4356-x (PMC6704683; doi:10.1186/s12879-019-4356-x)
Supplement: Supplementary file 1 — Appendix A. Details of the general study methodology for the clinical trial. Appendix A.1. Trial study_Screening and enrolment. Appendix A.2. Trial study_Sampling. Appendix A.3. Trial study_ Initiation of study medication, safety monitoring, dose adjustment. Appendix A.4. Trial study_Emergency management. Appendix A.5. Trial study_Emergency unblinding procedure. Appendix A.6. Trial study_Additional study definitions. Appendix A.7. Trial study_Definitions for Clinical Adverse Event Grading in the trial (modified from CTCAE Version 4.03). Appendix A.8. Trial study_Definitions for Laboratory Adverse Event Grading in the trial (modified from CTCAE Version 4.03). Appendix B. Additional methods for the observational cohort study. Appendix B.1. Cohort study_Identification of study subjects. Appendix B.2. Cohort study_Data collection and data management. Appendix B.3. Cohort study_Statistical analysis. (ZIP 257 kb) [file 12879_2019_4356_MOESM1_ESM.zip › Appendix A.6 - Trial study_Additional study definitionsR4.docx]

**Appendix A.6: Trial study_Additional study definitions**

**Autonomic nervous system (ANS) dysregulation**: At least 2 of the following features: heart rate of 150-170 beats/min, systolic blood pressure variability with absolute values higher than the 95th percentile for age, gender and height, profuse sweating, mottled skin, respiratory abnormalities, and hyperglycemia.

**The following definitions apply for the inclusion/exclusion criteria:**

**Tachypnea**: respiratory rate

- 6 – 12 months: ≥50
- 13 – 72 months: ≥40
- 6 – 12 years: ≥ 30
- 13 – 15 years: ≥25

**Refractory Fever:** Core temperature > 40^o^C for at least 4 hours despite antipyretics

**Hyperglycemia**: >150 mg/dl (8.3 mmol/l) on a random test, or >126 mg/dl (7 mmol/l) on a fasting test, taken at least 4 hours after feeding.

**Respiratory distress:** if the patient has any one of the following findings:

- Tachypnea
- Irregular breathing
- Wheeze
- Stridor
- Cheyne-Stokes breathing
- Gasp
- Apnoeic episodes

**Cardiac arrhythmia:**

- Sinus tachycardia is not an exclusion criterion, but any other cardiac arrhythmia is, including AV block (Grades I-III) or QT prolonged >0.48 ms

**Acute Renal failure:**

- Serum creatinine > 2mg/dl (176 μmol/l) or urine output < 1ml/kg/hr for 4 hours or more

**Prolongation of existing hospitalization:** duration of hospitalization > 14 days
