# Supplementary material for: Assessing the efficacy and safety of magnesium sulfate for management of autonomic nervous system dysregulation in Vietnamese children with severe hand foot and mouth disease
Source: BMC Infect Dis. 2019 Aug 22;19:737. doi: 10.1186/s12879-019-4356-x (PMC6704683; doi:10.1186/s12879-019-4356-x)
Supplement: Supplementary file 1 — Appendix A. Details of the general study methodology for the clinical trial. Appendix A.1. Trial study_Screening and enrolment. Appendix A.2. Trial study_Sampling. Appendix A.3. Trial study_ Initiation of study medication, safety monitoring, dose adjustment. Appendix A.4. Trial study_Emergency management. Appendix A.5. Trial study_Emergency unblinding procedure. Appendix A.6. Trial study_Additional study definitions. Appendix A.7. Trial study_Definitions for Clinical Adverse Event Grading in the trial (modified from CTCAE Version 4.03). Appendix A.8. Trial study_Definitions for Laboratory Adverse Event Grading in the trial (modified from CTCAE Version 4.03). Appendix B. Additional methods for the observational cohort study. Appendix B.1. Cohort study_Identification of study subjects. Appendix B.2. Cohort study_Data collection and data management. Appendix B.3. Cohort study_Statistical analysis. (ZIP 257 kb) [file 12879_2019_4356_MOESM1_ESM.zip › Appendix A.7 - Trial_Definitions for Clinical Adverse EventR4.docx]

**Appendix A.7: Trial study_Definitions for Clinical Adverse Event Grading in the trial (modified from CTCAE Version 4.03)**

|  | Grade | | | | |
| --- | --- | --- | --- | --- | --- |
| Adverse Event | 1 | 2 | 3 | 4 | 5 |
| 1. General | | | | | |
| Fever after enrolment  (core Temp) | 39 < 40.9 °C | 40-41 °C without any new CNS problem | 40-41 °C with new CNS problem | > 41 °C with new CNS problem or >42 °C for over 1 hour |  |
| Flushing | Asymptomatic, no intervention needed | Moderate, requiring symptomatic Rx only, e.g. antihistamines | Associated with hypotension and/or tachycardia | - | - |
| Mouth or Skin lesion | New lesions noted | - | - | - | - |
| Profuse sweating | Local | General | - | - | - |
| Mottled skin | Local | General | - | - | - |
| 2. Gastrointestinal disorders | | | | | |
| Vomiting | 1 - 2 times per day | 3 - 5 times per day | >= 6 times per day | - | - |
| Diarrhea | 1 - 3 loose stools per day | 4 – 6 loose stools per day | >=7 loose stools per day | Life-threatening with signs of dehydration. | Death |
| Liver palpable | Asymptomatic | Painful | - | - | - |
| Upper GI Bleeding | Occasional streaks of blood in vomit | Persistent blood but no clinical problems | Blood in vomit, needing Tx but not urgent | Life-threatening and urgent transfusion needed | Death |
| Lower GI Bleeding | Occasional streaks of blood in stool | Persistent blood but no clinical problems | Blood in stool, needing Tx but not urgent | Life-threatening and urgent transfusion needed | Death |
| 3. Cardiac disorders | | | | | |
| Cardiac arrest | - | - | - | Life-threatening, and urgent intervention indicated | Death |
| Atrioventricular block complete | - | No hemodynamic effect and resolves after stopping study drug | Any hemodynamic effects, even if these resolve after stopping study drug. Advice from cardiologist, but not urgent | Life-threatening with hemodynamic compromise, and requiring immediate intervention eg urgent pacing wire | Death |
| Atrioventricular block first degree | - | Asymptomatic, no intervention | - | - | - |
| 2nd Degree (Mobitz Type I or II) atrioventricular block |  | Asymptomatic, no intervention | Hemodynamic effects, requiring advice from cardiologist, not urgent | Life-threatening with hemodynamic compromise, and requiring immediate intervention | Death |
| QT interval (corrected for heart rate)* | QTc 450 - 480 ms | QTc 481 - 500 ms | QTc >= 501 ms on at least two separate ECGs | QTc >= 501 or >60 ms change from baseline and Torsade de pointes or polymorphic VT or other signs/symptoms of serious arrhythmia | Death |
| Other serious rhythm disturbance (on the monitor or a formal ECG) | - | Occasional atrial or ventricular ectopics, no intervention | Persistent atrial or ventricular ectopics, causing hemodynamic effects but resolving after withdrawal of study drug. AF not causing hemodynamic compromise or thrombotic / embolic complications. | Atrial or ventricular ectopics not responding to withdrawal of study drug. VT or VF. AF if associated with any thrombotic / embolic complication. | Death |
| Hypotension  (Systolic BP <70+2Xage) |  | Transient hypotension: close observation, no intervention, resolves within 15 minutes | Medical intervention required but not urgent | Life-threatening, and urgent intervention indicated | Death |
| Hypertensive emergency | - | - | - |  |  |
| 4. Respiratory disorders | | | | | |
| Hypoxia | - | Intermittent O_2_ saturation <92%, brief, no respiratory symptoms | Persistent O_2_ saturation <92%, despite nasal O_2_, but resolves in < 60 minutes | Decreased O_2_ saturation <92 %, failing to improve with nasal O_2_, and persisting for > 60 minutes requiring ventilatory support . | Death |
| Irregular breathing | Irregular breathing occurring transiently and no O_2_ indicated | Irregular breathing is persistent but SpO_2_ >92% when checked in air. | Irregular breathing and SpO_2_ < 92% without O_2_ | Life-threatening, needing urgent intervention, e.g. intubation or ventilatory support | Death |
| Stridor | - | Stridor occurs transiently but SpO_2_ >92% when checked in air. | Stridor occurs and SpO_2_ <92% without O_2_ | Life-threatening, needing urgent intervention, e.g. intubation or ventilatory support | Death |
| Respiratory retractions | - | Retractions occur transiently but SpO_2_ >92% when checked in air. | Retractions occur and SpO_2_ <92% without O_2_ | Life-threatening, needing urgent intervention, e.g. intubation or ventilatory support | Death |
| Apnea/Cheyne Stokes/ Gasps | - | - | - | Life-threatening, needing urgent intervention, e.g. intubation or ventilatory support | Death |
| Pulmonary edema |  |  | Severe dyspnea or dyspnea at rest; O_2_ indicated; | Life-threatening respiratory compromise; urgent intervention or intubation with ventilatory support indicated | Death |
| 5. Nervous system disorders | | | | | |
| Coma | GCS ≥ 14 | 11 ≤ GCS ≤ 13 | 9 ≤ GCS ≤ 10 | GCS ≤ 8 | Death |
| Convulsions |  | Brief (< 1 minute) generalized seizure | Multiple seizures requiring drug Rx but not respiratory support | Prolonged repetitive seizures, poor response to treatment, requiring respiratory support (CPAP or IPPV) | Death |
| Pupils reactive | - | - | Unreactive or > 3mm in one eye | Unreactive or > 3mm in both eyes |  |
| Headache | Mild pain, no medication needed | Moderate pain requiring paracetamol intermittently | Severe pain, requiring regular or continuous paracetamol. | - | - |
| Lethargy | Intermittent reduced alertness and awareness (not related to a convulsion) | Persistently reduced alertness and awareness for several hours | - | - | - |
| Irritability | Intermittent | Persistent | - | - | - |
| Myoclonic jerks | ≤ 2 times/ day | 3 - 10 times/ day | > 10 times /day | - | - |
| Ataxia/tremor | Intermittent | Persistent | - | - | - |
| Nystagmus/eye wandering | Intermittent | Persistent | - | - | - |
| Diminished DTRs | Asymptomatic, reduced but still present | Complete loss of DTRs, no resp. distress | Complete loss of DTRs, with resp. distress | - | - |
| Limb paralysis (severity assessment at discharge) | Minor weakness, only detectable on examination | Partial weakness of 1 limb, but able to function (age appropriate) with support | Partial weakness of >1 limb, but able to function (age appropriate) with support | Profound weakness, dependent on support from carers |  |
| Cranial nerve paralysis (severity assessment at discharge) | - | Observation only, no clinical effects | With clinical effects, eg. Swallowing difficulty, medical management only | With severe clinical consequences requiring invasive management eg. Tracheostomy | - |
| 6. Renal disorders | | | | | |
| Reduced urine output | - | Transient reduction in urine output, improving within 4 hours without Rx |  |  | Death |
| Haematuria | - | Visible blood but no clinical problems | Needing Tx but not urgent | Life-threatening and urgent transfusion needed | Death |
| Urinary retention | Temporary, responding to physical stimulation of bladder | Persistent requiring catheterisation |  |  |  |
| 7. Other | | | | | |
| Cough, coryza, conjuncitivitis | Mild symptoms, no treatment needed | - | - | - | - |
| Abdominal Pain | Mild pain, no medication needed | Moderate pain requiring paracetamol intermittently | Severe pain, requiring regular or continuous paracetamol. | - | - |
| Abdominal distension | Mild, visible but asymptomatic | Causing some minor distress but no intervention needed | Causing distress sufficient to need rectal catheter insertion | Resulting in perforation | - |
|  |  |  |  |  |  |
