# Supplementary material for: Assessing the efficacy and safety of magnesium sulfate for management of autonomic nervous system dysregulation in Vietnamese children with severe hand foot and mouth disease
Source: BMC Infect Dis. 2019 Aug 22;19:737. doi: 10.1186/s12879-019-4356-x (PMC6704683; doi:10.1186/s12879-019-4356-x)
Supplement: Supplementary file 1 — Appendix A. Details of the general study methodology for the clinical trial. Appendix A.1. Trial study_Screening and enrolment. Appendix A.2. Trial study_Sampling. Appendix A.3. Trial study_ Initiation of study medication, safety monitoring, dose adjustment. Appendix A.4. Trial study_Emergency management. Appendix A.5. Trial study_Emergency unblinding procedure. Appendix A.6. Trial study_Additional study definitions. Appendix A.7. Trial study_Definitions for Clinical Adverse Event Grading in the trial (modified from CTCAE Version 4.03). Appendix A.8. Trial study_Definitions for Laboratory Adverse Event Grading in the trial (modified from CTCAE Version 4.03). Appendix B. Additional methods for the observational cohort study. Appendix B.1. Cohort study_Identification of study subjects. Appendix B.2. Cohort study_Data collection and data management. Appendix B.3. Cohort study_Statistical analysis. (ZIP 257 kb) [file 12879_2019_4356_MOESM1_ESM.zip › Appendix A.8 - Trial_Definitions for Laboratory AER4.docx]

**Appendix A.8: Trial study_Definitions for Laboratory Adverse Event Grading in the trial (modified from CTCAE Version 4.03)**

**A** laboratory abnormality is one that was: 1) not present at baseline, or 2) was present at baseline and has now worsened; 3) was present at baseline, improved for >24 hours, and has now recurred, ***If the value varies during any 24 hour period, the worst grade abnormality will be recorded.***

|  |  |  |  |  |  |
| --- | --- | --- | --- | --- | --- |
| **Abnormalities** | 1 | 2 | 3 | 4 | 5 |
| Acidosis | pH <7.37, but >=7.3 | - | pH <7.3 | Life-threatening consequences | Death |
| Alkalosis | pH >7.45, but <=7.5 | - | pH >7.5 | Life-threatening consequences | Death |
| Hemoglobin (Hgb) | 6months-2yrs: 10.0 - <10.5 g/dl  >2 yrs: 10.0 - < 11.5 g/dL | <10.0 - 8.0 g/dL | <8.0 g/dL | Life-threatening consequences urgent intervention indicated | Death |
| Platelet count decreased | <LLN - 75,000/mm3 | <75,000 - 50,000/mm3 | 50,000 - 25,000/mm3 | < 25,000/mm3 | - |
| Cardiac Troponin I increased* | >ULN - 2.5 x ULN | >2.5 x ULN - 5 x ULN | >5 x ULN - 10 x ULN | > 10 x ULN | - |
| Before 11Mar2015: ULN: 0.3 ng/l | > 0.3 - 0.75 | > 0.75 - 1.5 | >1.5 - 3 | > 3 |  |
| From 11Mar2015: ULN: 15.6 (female), 34.2 (male) pg/l | >15.6 – 39 (female); 34.2-85.5 (male) | > 39– 78 (female); 85.5-171 (male) | >79– 156 (female); 171 -342 (male) | >156 (female); 342 (male) |  |
| CK-MB  ULN: 24 UI/l | >ULN - 2.5 x ULN  (> 24- 60) | >2.5 x ULN - 5 x ULN  (> 60-120) | >5 x ULN - 10 x ULN  (>120 – 240) | > 10 x ULN  (>240) | - |
| Creatinine increased  ULN: <4yr : 42.2, / 4-10yr : 52.2 / >10-14 yr: 77.8 umol/l | >ULN -1.5 x ULN | >1.5 - 3.0 x ULN | >3.0 - 6.0 x ULN | >6.0 x ULN | - |
| Hyponatremia  LLN: 135 mmol/l | <135 - 130 mmol/L | - | <130 - 120 mmol/L | <120 mmol/L | Death |
| Hypernatremia  ULN: 145 mmol/l | >145 - 150 mmol/L | >150 - 155 mmol/L | >155 - 160 mmol/L | >160 mmol/L | Death |
| Hyperkalemia | >5 - 5.5 mmol/L | >5.5 - 6.0 mmol/L | >6.0 - 7.0 mmol/L | >7.0 mmol/L | Death |
| Hypokalemia | <3.5 - 3.0 mmol/L | - | <3.0 - 2.5 mmol/L | <2.5 mmol/L | Death |
| Hypermagnesemia | >1 -1.23 mmol/L | ->1.23 – 2.5 mmol/L | >2.5 – 3.3 mmol/L | >3.30 mmol/L | Death |
| Hypocalcemia: ULN: 2.2 mmol/l  (~ Ionized calcium: 1.1) | Serum calcium of <2.2 - 2 mmol/L or  Ionized calcium of <1.1 - 1 mmol/l | Serum calcium of <2.0 - 1.8 mmol/L  Ionized calcium of <1 - 0.9 mmol/l | Serum calcium of <1.8 - 1.5 mmol/L or  Ionized calcium of <0.9 - 0.75 mmol/l | Serum calcium of <1.5 mmol/L or  Ionized calcium of <0.75 mmol/l | Death |
| Hypoglycemia | <60 - 55 mg/dL or  < 3.33 – 3.05 mmol/l | <55 - 40 mg/dL or  < 3.05 – 2.2 mmol/l | <40 - 30 mg/dL or  < 2.2 – 1.66 mmol/l | <30 mg/dL or  < 1.66 mmol/l | Death |
| Hyperglycemia: ULN: 120 mg/dl or 6.7 mmol/l | Fasting glucose > 120 - 160 mg/dL or  > 6.7 - 8.88 mmol/l | Fasting glucose >160 - 250 mg/dL or > 8.88 – 13.88 mmol/l | Fasting glucose >250 - 500 mg/dL or > 13.88 – 27.8 mmol/l | Fasting glucose >500 mg/dL or  > 27.8 mmol/l | Death |

* Note change in lab procedures on 11th Mar 2015, with new normal ranges for Troponin
