# Supplementary material for: Assessing the efficacy and safety of magnesium sulfate for management of autonomic nervous system dysregulation in Vietnamese children with severe hand foot and mouth disease
Source: BMC Infect Dis. 2019 Aug 22;19:737. doi: 10.1186/s12879-019-4356-x (PMC6704683; doi:10.1186/s12879-019-4356-x)
Supplement: Supplementary file 1 — Appendix A. Details of the general study methodology for the clinical trial. Appendix A.1. Trial study_Screening and enrolment. Appendix A.2. Trial study_Sampling. Appendix A.3. Trial study_ Initiation of study medication, safety monitoring, dose adjustment. Appendix A.4. Trial study_Emergency management. Appendix A.5. Trial study_Emergency unblinding procedure. Appendix A.6. Trial study_Additional study definitions. Appendix A.7. Trial study_Definitions for Clinical Adverse Event Grading in the trial (modified from CTCAE Version 4.03). Appendix A.8. Trial study_Definitions for Laboratory Adverse Event Grading in the trial (modified from CTCAE Version 4.03). Appendix B. Additional methods for the observational cohort study. Appendix B.1. Cohort study_Identification of study subjects. Appendix B.2. Cohort study_Data collection and data management. Appendix B.3. Cohort study_Statistical analysis. (ZIP 257 kb) [file 12879_2019_4356_MOESM1_ESM.zip › Appendix B.1 - Cohort study_Identification of subjectsR4.docx]

### Appendix B.1: Cohort study_Identification of study subjects

The HTD main database records basic information on all patients admitted to the hospital, including information on all medications prescribed. From this database one individual extracted the hospital file numbers of all children on PICU who received milrinone between January 2011 and December 2015. We then requested these files and checked each record to confirm that the patients had been admitted to PICU with a clinical diagnosis of HFMD, and that they had not been enrolled in the MgSO_4_ RCT.

We determined which patients did not respond to milrinone according to the following criteria, and also the date/time when they reached this severity level:

a) SBP persisting above Stage 2 hypertension (VN MoH guidelines) for at least 60 minutes after commencing maximum dose milrinone, i.e. 0.7 µg/kg/minute;

b) SBP increasing rapidly (by more than 10 mm Hg above the MoH threshold) after reaching a dose of at least 0.6 µg/kg/minute milrinone, or within 30 minutes of moving up to 0.7 µg/kg/minute.

We also checked that the patients a) had not received fluid resuscitation or inotropes such as dopamine, adrenaline or noradrenaline during this time-period or previously during the hospital admission, and b) had not needed any other supportive therapy such as ventilation or haemofiltration during this time-period or previously.

We selected all cases who fulfilled these criteria for detailed file review and data extraction. For the MgSO_4_ group the date/time of starting the drug was taken as T=0, while for the control subjects T=0 was imputed as described below.
