# Supplementary material for: Assessing the efficacy and safety of magnesium sulfate for management of autonomic nervous system dysregulation in Vietnamese children with severe hand foot and mouth disease
Source: BMC Infect Dis. 2019 Aug 22;19:737. doi: 10.1186/s12879-019-4356-x (PMC6704683; doi:10.1186/s12879-019-4356-x)
Supplement: Supplementary file 1 — Appendix A. Details of the general study methodology for the clinical trial. Appendix A.1. Trial study_Screening and enrolment. Appendix A.2. Trial study_Sampling. Appendix A.3. Trial study_ Initiation of study medication, safety monitoring, dose adjustment. Appendix A.4. Trial study_Emergency management. Appendix A.5. Trial study_Emergency unblinding procedure. Appendix A.6. Trial study_Additional study definitions. Appendix A.7. Trial study_Definitions for Clinical Adverse Event Grading in the trial (modified from CTCAE Version 4.03). Appendix A.8. Trial study_Definitions for Laboratory Adverse Event Grading in the trial (modified from CTCAE Version 4.03). Appendix B. Additional methods for the observational cohort study. Appendix B.1. Cohort study_Identification of study subjects. Appendix B.2. Cohort study_Data collection and data management. Appendix B.3. Cohort study_Statistical analysis. (ZIP 257 kb) [file 12879_2019_4356_MOESM1_ESM.zip › Appendix B.2 - Cohort study_Data collection and managementR4.docx]

### Appendix B.2: Cohort study_Data collection and data management

We developed a specific case report form (CRF) for this study focused on documenting all available information on cardiovascular parameters in these patients. The CRF included baseline information from T=0 and then hourly nursing observations for a minimum of 24 hours from that time, then with reduced frequency for up to 72 hours or until recordings in the hospital files stopped. In addition data for up to 6 hours prior to T-0 were extracted from the files, provided the patient was in PICU during this period. The database also included once daily assessments during the PICU stay, with information on clinical signs, particularly neurological features and cardiovascular and respiratory manifestations, collected from the routine daily ward round documentation. Information on ANS manifestations such as mottled skin, sweating etc. was not systematically recorded in the hospital files during this period, but any information noted in the files was collected
