# Supplementary material for: Assessing the efficacy and safety of magnesium sulfate for management of autonomic nervous system dysregulation in Vietnamese children with severe hand foot and mouth disease
Source: BMC Infect Dis. 2019 Aug 22;19:737. doi: 10.1186/s12879-019-4356-x (PMC6704683; doi:10.1186/s12879-019-4356-x)
Supplement: Supplementary file 1 — Appendix A. Details of the general study methodology for the clinical trial. Appendix A.1. Trial study_Screening and enrolment. Appendix A.2. Trial study_Sampling. Appendix A.3. Trial study_ Initiation of study medication, safety monitoring, dose adjustment. Appendix A.4. Trial study_Emergency management. Appendix A.5. Trial study_Emergency unblinding procedure. Appendix A.6. Trial study_Additional study definitions. Appendix A.7. Trial study_Definitions for Clinical Adverse Event Grading in the trial (modified from CTCAE Version 4.03). Appendix A.8. Trial study_Definitions for Laboratory Adverse Event Grading in the trial (modified from CTCAE Version 4.03). Appendix B. Additional methods for the observational cohort study. Appendix B.1. Cohort study_Identification of study subjects. Appendix B.2. Cohort study_Data collection and data management. Appendix B.3. Cohort study_Statistical analysis. (ZIP 257 kb) [file 12879_2019_4356_MOESM1_ESM.zip › Appendix B.3 - Cohort study_Statistical analysisR4.docx]

### Appendix B.3: Cohort study_Statistical analysis

Patients who received MgSO_4_ for hypertension were considered as the exposed patients, while those who did not receive MgSO_4_ but may have received alternative second line therapy, were considered as unexposed and formed the control group.

**Major events:** We assessed a composite endpoint comprising progression to any major clinical event within the first 72 hours after T=0; the endpoints were shock, need for inotropic support, respiratory compromise requiring ventilation, or death. Comparison between the groups was based on log-binomial regression model with adjustment for age and day of illness at study entry. However, there were no deaths and very few major events in either patient group. The difference in duration of milrinone therapy and of hospitalization after T=0 were assessed using linear regression.

**Comparison of hemodynamic parameters between treatment groups:** We wanted to compare the magnitude and time course of hemodynamic stabilization over the 24 hours after T=0 in the two groups to see if blood pressure was more effectively controlled in the MgSO_4_ group. For this analysis we focused on the SBP and the mean arterial pressure (MAP) calculated in a standard way as follows: [SBP+2(DBP)]/3.

Examination of the MgSO_4_ group data showed that the actual time of starting MgSO_4_ in this group was quite variable in relation to the time when they became eligible as defined in the selection criteria described earlier. A number of factors may have influenced this clinical decision-making including the age of the patient, the rapidity of the deterioration in blood pressure, and the experience of the clinician involved. For this detailed analysis of hemodynamic parameters and so as to allow for the intrinsic variability in the initiation time in the MgS0_4_ group, we developed a prediction model for MgSO_4_ initiation after becoming eligible. This model was then used to create multiple datasets that imputed time points for when MgSO_4_ might have been initiated in the control group.

This prediction/imputation model is a logistic regression model based on three factors:

a) the difference between the current SBP and the age-dependent cut-off for Stage 2 hypertension

b) the difference between the current SBP and the previous SBP value

c) the current dose of milrinone

For control patients, at each time-point when the SBP was measured and was larger than the age-dependent cut-off, a probability of initiating MgSO_4_ was estimated from the imputation model. For each patient, the imputed time of MgSO_4_ initiation was the first instance when MgSO_4_ would be used, based on random assignment using a Bernoulli distribution where the probability of success is the probability of initiating MgSO_4_ estimated from the imputation model. A patient was considered as not receiving MgSO_4_ if it was not initiated at all time-points based on these probability models. This procedure was repeated 20 times for each patient to create 20 imputed datasets.

Although MgSO_4_ was initiated at Stage 2 hypertension in the exposed group after failure with milrinone, this is a rather high BP level and Stage 1 hypertension is more relevant as a clinical threshold for concern and potential intervention. Therefore, we examined the area under the curve (AUC) for all SBP and the MAP values above the appropriate age-dependent Stage 1 hypertension threshold during the first 24 hours in the two groups, comparing values after actual T=0 in the exposed MgSO_4_ group and presumptive time points for initiating MgSO_4_ in 20 imputation datasets in the control group. As AUCs for SBP and MAP were skewed to the right, we log10-transformed these variables before applying regression analysis. We also added 1 to each MAP before transformation to avoid taking logarithm of zero.

To combined results across imputed datasets, we used both the conventional Rubin’s rule and a modified rule. The modified rule is based on the weighted mean and weighted variance, where the weight is the ratio between the size of each imputed dataset and the total size of all 20 datasets. Details of the calculation are given below:

$m:$ number of imputed datasets

$Q_{i}:$ mean estimate from imputed dataset $i (i=1, \ldots,m)$

$U_{i}:$ variance estimate from imputed dataset $i$

$n_{i}:$ size of imputed dataset $i$

$w_{i}:$ weight for estimates from imputed dataset $i$ $(w_{i}=\frac{n_{i}}{\sum n_{i}})$

$$V=\sum{(w_{i})}^{2}$$

- Pooled mean estimate $Q$

$$Q=\sum{w_{i}\times Q}_{i}$$

- Pooled variance estimate $T$

$$T=\sum w_{i}\times U_{i}+ \left( 1+\frac{1}{m} \right)\left( \frac{1}{1-V}\sum w_{i}\times{(Q_{i}-Q)}^{2} \right)$$

When weight is fixed as $\frac{1}{m}$, we will obtain the Rubin’s rule.
