# Supplementary material for: Assessing the efficacy and safety of magnesium sulfate for management of autonomic nervous system dysregulation in Vietnamese children with severe hand foot and mouth disease
Source: BMC Infect Dis. 2019 Aug 22;19:737. doi: 10.1186/s12879-019-4356-x (PMC6704683; doi:10.1186/s12879-019-4356-x)
Supplement: Supplementary file 2 — Table S1. Brief Summary of the Vietnamese MoH Classification for HFMD. (DOCX 81 kb) [file 12879_2019_4356_MOESM2_ESM.docx]

**Additional file 2: Table S1**: Brief Summary of the Vietnamese MoH Classification for HFMD*

| Grade | Clinical manifestation |
| --- | --- |
| 1 | Uncomplicated HFMD including skin lesions and/or mouth ulcers |
| 2a | Possible neurological involvement including myoclonic jerks observed by the family or confusion or high persistent fever. |
| 2b | Neurological involvement including witnessed myoclonic jerks or cerebellar signs or paralysis or refractory fever. |
| 3 | Autonomic nervous system dyregulation including hypertension, persistent tachycardia, respiratory abnormalities (tachypnea or labored breathing), or coma. |
| 4 | Cardiopulmonary failure including hypotension/shock, pulmonary edema or heart failure. |

*: The full details of the VN MoH HFMD classification are described in the published protocol
