# Supplementary material for: Assessing the efficacy and safety of magnesium sulfate for management of autonomic nervous system dysregulation in Vietnamese children with severe hand foot and mouth disease
Source: BMC Infect Dis. 2019 Aug 22;19:737. doi: 10.1186/s12879-019-4356-x (PMC6704683; doi:10.1186/s12879-019-4356-x)
Supplement: Supplementary file 3 — Table S2. Comparison of Bayley-III neurodevelopmental assessments 6 months after discharge for the clinical trial participants. (DOCX 78 kb) [file 12879_2019_4356_MOESM3_ESM.docx]

Additional file 3: Table S2: Comparison of Bayley-III neurodevelopmental assessments 6 months after discharge between the two treatment arms in the clinical trial

|  | n ^#^ | Placebo  (N=12) | n | MgSO_4_  (N=14) | Unadjusted mean difference | p-value | Adjusted mean difference* | p-value |
| --- | --- | --- | --- | --- | --- | --- | --- | --- |
| CS | 10 | 2.5 (0.1, 5.5) | 12 | 1.4 (-1.3, 4.1) | -0.73 (-2.11, 0.65) | 0.254 | -0.28 (-1.38, 0.83) | 0.532 |
| RC | 10 | 1.1 (-5.3, 8.8) | 12 | 1.5 (-0.6, 5.2) | 0.26 (-2.12, 2.63) | 0.813 | 0.61 (-1.40, 2.61) | 0.455 |
| EC | 10 | 1.2 (-1.0, 3.0) | 12 | 1.6 (-1.0, 5.1) | 0.18 (-1.13, 1.49) | 0.765 | 0.61 (-0.65, 1.86) | 0.238 |
| FM | 10 | 2.6 (0.2, 5.5) | 12 | 1.9 (-0.6, 3.5) | -0.92 (-2.18, 0.34) | 0.876 | -0.79 (-2.12, 0.54) | 0.150 |
| GM | 10 | 1.9 (0.0, 4.0) | 12 | 2.2 (0.4, 3.8) | 0.07 (-0.91, 1.05) | 0.876 | 0.37 (-0.59, 1.33) | 0.341 |

CS= Cognitive Scale; RC: Receptive Communication; EC= Expressive Communication; FM= Fine Motor; GM= Gross Motor

Summary statistic is the median (range) of Z-scores

#: 2 children older than 48 months were assessed by Movement ABC-2 tool; 1 child could not be assessed as the mother tongue was Chinese, 1 child would not cooperate with the test.

*: Mean differences of Z-scores were adjusted for sex, age, and maternal education level
