# Supplementary material for: Assessing the efficacy and safety of magnesium sulfate for management of autonomic nervous system dysregulation in Vietnamese children with severe hand foot and mouth disease
Source: BMC Infect Dis. 2019 Aug 22;19:737. doi: 10.1186/s12879-019-4356-x (PMC6704683; doi:10.1186/s12879-019-4356-x)
Supplement: Supplementary file 4 — Table S3. AUCs of systolic blood pressure above the stage 1 hypertension cut-off, comparisons between groups who received MgSO4 and those who did not, for each imputed dataset plus the overall pooling. (DOCX 93 kb) [file 12879_2019_4356_MOESM4_ESM.docx]

Additional file 4: Table S3: Log 10 AUCs of systolic blood pressure above the stage 1 hypertension cut-off, comparisons between groups that received MgSO_4_ and those that did not, for each imputed dataset.

Note: The number of patients in the control group varied between the imputed datasets as patients for whom the time to initiate MgSO_4_ was undetermined (i.e. MgSO_4_ was not given based on the prediction/imputation model) were excluded.

| **Dataset** | **n** | **Control** | **n** | **MgSO4** | **Variables** | **Estimate** | **Lower 95%CI** | **Upper 95%CI** | **p** |
| --- | --- | --- | --- | --- | --- | --- | --- | --- | --- |
| Imputation 1 |  |  |  |  |  |  |  |  |  |
| Median (range) | 10 | 2.50 (2.27 - 2.70) | 33 | 2.50 (2.28 - 2.66) | Intercept | 2.08 | 1.85 | 2.32 | <0.001 |
| Mean (SD) | 10 | 2.45 (0.36) | 33 | 2.42 (0.33) | MgSO4 group | -0.02 | -0.21 | 0.18 | 0.855 |
|  |  |  |  |  | Initial SBP above cut-off | 0.01 | 0.01 | 0.02 | <0.001 |
| Imputation 2 |  |  |  |  |  |  |  |  |  |
| Median (range) | 10 | 2.52 (2.21 - 2.70) | 33 | 2.50 (2.28 - 2.66) | Intercept | 2.04 | 1.81 | 2.26 | <0.001 |
| Mean (SD) | 10 | 2.41 (0.41) | 33 | 2.42 (0.33) | MgSO4 group | -0.01 | -0.21 | 0.19 | 0.923 |
|  |  |  |  |  | Initial SBP above cut-off | 0.01 | 0.01 | 0.02 | <0.001 |
| Imputation 3 |  |  |  |  |  |  |  |  |  |
| Median (range) | 12 | 2.48 (2.31 - 2.68) | 33 | 2.50 (2.28 - 2.66) | Intercept | 2.15 | 1.95 | 2.34 | <0.001 |
| Mean (SD) | 12 | 2.44 (0.33) | 33 | 2.42 (0.33) | MgSO4 group | -0.08 | -0.26 | 0.1 | 0.386 |
|  |  |  |  |  | Initial SBP above cut-off | 0.01 | 0.01 | 0.02 | <0.001 |
| Imputation 4 |  |  |  |  |  |  |  |  |  |
| Median (range) | 11 | 2.52 (2.28 - 2.68) | 33 | 2.50 (2.28 - 2.66) | Intercept | 2.12 | 1.92 | 2.33 | <0.001 |
| Mean (SD) | 11 | 2.46 (0.30) | 33 | 2.42 (0.33) | MgSO4 group | -0.06 | -0.24 | 0.11 | 0.485 |
|  |  |  |  |  | Initial SBP above cut-off | 0.01 | 0.01 | 0.02 | <0.001 |
| Imputation 5 |  |  |  |  |  |  |  |  |  |
| Median (range) | 9 | 2.53 (2.34 - 2.71) | 33 | 2.50 (2.28 - 2.66) | Intercept | 2.19 | 1.98 | 2.41 | <0.001 |
| Mean (SD) | 9 | 2.50 (0.32) | 33 | 2.42 (0.33) | MgSO4 group | -0.13 | -0.33 | 0.06 | 0.193 |
|  |  |  |  |  | Initial SBP above cut-off | 0.01 | 0.01 | 0.02 | <0.001 |
| Imputation 6 |  |  |  |  |  |  |  |  |  |
| Median (range) | 10 | 2.51 (2.34 - 2.70) | 33 | 2.50 (2.28 - 2.66) | Intercept | 2.15 | 1.93 | 2.37 | <0.001 |
| Mean (SD) | 10 | 2.52 (0.25) | 33 | 2.42 (0.33) | MgSO4 group | -0.08 | -0.26 | 0.1 | 0.39 |
|  |  |  |  |  | Initial SBP above cut-off | 0.01 | 0.01 | 0.02 | <0.001 |
| Imputation 7 |  |  |  |  |  |  |  |  |  |
| Median (range) | 11 | 2.52 (2.29 - 2.69) | 33 | 2.50 (2.28 - 2.66) | Intercept | 2.09 | 1.87 | 2.3 | <0.001 |
| Mean (SD) | 11 | 2.44 (0.35) | 33 | 2.42 (0.33) | MgSO4 group | -0.04 | -0.23 | 0.14 | 0.659 |
|  |  |  |  |  | Initial SBP above cut-off | 0.01 | 0.01 | 0.02 | <0.001 |
| Imputation 8 |  |  |  |  |  |  |  |  |  |
| Median (range) | 12 | 2.43 (2.22 - 2.68) | 33 | 2.50 (2.28 - 2.66) | Intercept | 2.03 | 1.81 | 2.25 | <0.001 |
| Mean (SD) | 12 | 2.40 (0.36) | 33 | 2.42 (0.33) | MgSO4 group | 0.01 | -0.17 | 0.19 | 0.895 |
|  |  |  |  |  | Initial SBP above cut-off | 0.01 | 0.01 | 0.02 | <0.001 |
| Imputation 9 |  |  |  |  |  |  |  |  |  |
| Median (range) | 12 | 2.52 (2.27 - 2.67) | 33 | 2.50 (2.28 - 2.66) | Intercept | 2.11 | 1.9 | 2.31 | <0.001 |
| Mean (SD) | 12 | 2.47 (0.30) | 33 | 2.42 (0.33) | MgSO4 group | -0.05 | -0.22 | 0.12 | 0.538 |
|  |  |  |  |  | Initial SBP above cut-off | 0.01 | 0.01 | 0.02 | <0.001 |
| Imputation 10 |  |  |  |  |  |  |  |  |  |
| Median (range) | 10 | 2.47 (2.34 - 2.70) | 33 | 2.50 (2.28 - 2.66) | Intercept | 2.05 | 1.82 | 2.28 | <0.001 |
| Mean (SD) | 10 | 2.45 (0.41) | 33 | 2.42 (0.33) | MgSO4 group | -0.04 | -0.23 | 0.16 | 0.705 |
|  |  |  |  |  | Initial SBP above cut-off | 0.01 | 0.01 | 0.02 | <0.001 |
| Imputation 11 |  |  |  |  |  |  |  |  |  |
| Median (range) | 12 | 2.46 (2.29 - 2.68) | 33 | 2.50 (2.28 - 2.66) | Intercept | 2.08 | 1.87 | 2.29 | <0.001 |
| Mean (SD) | 12 | 2.43 (0.32) | 33 | 2.42 (0.33) | MgSO4 group | -0.02 | -0.19 | 0.16 | 0.852 |
|  |  |  |  |  | Initial SBP above cut-off | 0.01 | 0.01 | 0.02 | <0.001 |
| Imputation 12 |  |  |  |  |  |  |  |  |  |
| Median (range) | 12 | 2.50 (2.32 - 2.67) | 33 | 2.50 (2.28 - 2.66) | Intercept | 2.19 | 1.99 | 2.39 | <0.001 |
| Mean (SD) | 12 | 2.47 (0.33) | 33 | 2.42 (0.33) | MgSO4 group | -0.1 | -0.29 | 0.08 | 0.274 |
|  |  |  |  |  | Initial SBP above cut-off | 0.01 | 0.01 | 0.02 | <0.001 |
| Imputation 13 |  |  |  |  |  |  |  |  |  |
| Median (range) | 10 | 2.52 (2.35 - 2.70) | 33 | 2.50 (2.28 - 2.66) | Intercept | 2.17 | 1.97 | 2.38 | <0.001 |
| Mean (SD) | 10 | 2.51 (0.27) | 33 | 2.42 (0.33) | MgSO4 group | -0.12 | -0.29 | 0.06 | 0.209 |
|  |  |  |  |  | Initial SBP above cut-off | 0.01 | 0.01 | 0.02 | <0.001 |
| Imputation 14 |  |  |  |  |  |  |  |  |  |
| Median (range) | 12 | 2.45 (2.30 - 2.68) | 33 | 2.50 (2.28 - 2.66) | Intercept | 2.09 | 1.89 | 2.29 | <0.001 |
| Mean (SD) | 12 | 2.46 (0.27) | 33 | 2.42 (0.33) | MgSO4 group | -0.04 | -0.2 | 0.13 | 0.667 |
|  |  |  |  |  | Initial SBP above cut-off | 0.01 | 0.01 | 0.02 | <0.001 |
| Imputation 15 |  |  |  |  |  |  |  |  |  |
| Median (range) | 12 | 2.51 (2.28 - 2.68) | 33 | 2.50 (2.28 - 2.66) | Intercept | 1.98 | 1.71 | 2.24 | <0.001 |
| Mean (SD) | 12 | 2.34 (0.62) | 33 | 2.42 (0.33) | MgSO4 group | 0 | -0.24 | 0.24 | 0.992 |
|  |  |  |  |  | Initial SBP above cut-off | 0.02 | 0.01 | 0.02 | <0.001 |
| Imputation 16 |  |  |  |  |  |  |  |  |  |
| Median (range) | 12 | 2.51 (2.34 - 2.68) | 33 | 2.50 (2.28 - 2.66) | Intercept | 2.1 | 1.89 | 2.31 | <0.001 |
| Mean (SD) | 12 | 2.42 (0.39) | 33 | 2.42 (0.33) | MgSO4 group | -0.04 | -0.23 | 0.15 | 0.646 |
|  |  |  |  |  | Initial SBP above cut-off | 0.01 | 0.01 | 0.02 | <0.001 |
| Imputation 17 |  |  |  |  |  |  |  |  |  |
| Median (range) | 10 | 2.52 (2.30 - 2.70) | 33 | 2.50 (2.28 - 2.66) | Intercept | 2.15 | 1.94 | 2.35 | <0.001 |
| Mean (SD) | 10 | 2.49 (0.29) | 33 | 2.42 (0.33) | MgSO4 group | -0.09 | -0.27 | 0.09 | 0.348 |
|  |  |  |  |  | Initial SBP above cut-off | 0.01 | 0.01 | 0.02 | <0.001 |
| Imputation 18 |  |  |  |  |  |  |  |  |  |
| Median (range) | 10 | 2.50 (1.95 - 2.70) | 33 | 2.50 (2.28 - 2.66) | Intercept | 1.98 | 1.75 | 2.21 | <0.001 |
| Mean (SD) | 10 | 2.35 (0.50) | 33 | 2.42 (0.33) | MgSO4 group | 0.02 | -0.19 | 0.22 | 0.868 |
|  |  |  |  |  | Initial SBP above cut-off | 0.02 | 0.01 | 0.02 | <0.001 |
| Imputation 19 |  |  |  |  |  |  |  |  |  |
| Median (range) | 12 | 2.48 (2.32 - 2.68) | 33 | 2.50 (2.28 - 2.66) | Intercept | 2.18 | 2.01 | 2.36 | <0.001 |
| Mean (SD) | 12 | 2.50 (0.23) | 33 | 2.42 (0.33) | MgSO4 group | -0.13 | -0.29 | 0.03 | 0.109 |
|  |  |  |  |  | Initial SBP above cut-off | 0.01 | 0.01 | 0.02 | <0.001 |
| Imputation 20 |  |  |  |  |  |  |  |  |  |
| Median (range) | 12 | 2.45 (2.34 - 2.66) | 33 | 2.50 (2.28 - 2.66) | Intercept | 2.12 | 1.92 | 2.31 | <0.001 |
| Mean (SD) | 12 | 2.46 (0.30) | 33 | 2.42 (0.33) | MgSO4 group | -0.07 | -0.24 | 0.1 | 0.43 |
|  |  |  |  |  | Initial SBP above cut-off | 0.01 | 0.01 | 0.02 | <0.001 |
