# Supplementary material for: Assessing the efficacy and safety of magnesium sulfate for management of autonomic nervous system dysregulation in Vietnamese children with severe hand foot and mouth disease
Source: BMC Infect Dis. 2019 Aug 22;19:737. doi: 10.1186/s12879-019-4356-x (PMC6704683; doi:10.1186/s12879-019-4356-x)
Supplement: Supplementary file 5 — Table S4. AUCs of mean arterial pressure (MAP) above the stage 1 hypertension cut-off, comparisons between groups who received MgSO4 and those who did not, for each imputed dataset plus the overall pooling. (DOCX 31 kb) [file 12879_2019_4356_MOESM5_ESM.docx]

Additional file 5: Table S4: Log 10 AUCs of mean arterial pressure (MAP) above the stage 1 hypertension cut-off, comparisons between groups that received MgSO_4_ and those that did not, for each imputed dataset.

Note: The number of patients in the control group varied between the imputed datasets as patients for whom the time to initiate MgSO_4_ was undetermined (i.e. MgSO_4_ was not given based on the prediction/imputation model) were excluded.

| **Dataset** | **n** | **Control** | **n** | **MgSO4** | **Variables** | **Estimate** | **Lower 95%CI** | **Upper 95%CI** | **p** |
| --- | --- | --- | --- | --- | --- | --- | --- | --- | --- |
| Imputation 1 |  |  |  |  |  |  |  |  |  |
| Median (range) | 10 | 1.69 (1.27 - 2.17) | 33 | 1.62 (1.39 - 1.86) | Intercept | 1.36 | 1.02 | 1.7 | <0.001 |
| Mean (SD) | 10 | 1.66 (0.77) | 33 | 1.54 (0.57) | MgSO4 group | -0.09 | -0.45 | 0.27 | 0.621 |
|  |  |  |  |  | Initial MAP above cut-off | 0.03 | 0.02 | 0.05 | <0.001 |
| Imputation 2 |  |  |  |  |  |  |  |  |  |
| Median (range) | 10 | 1.71 (1.38 - 2.21) | 33 | 1.62 (1.39 - 1.86) | Intercept | 1.46 | 1.13 | 1.78 | <0.001 |
| Mean (SD) | 10 | 1.70 (0.76) | 33 | 1.54 (0.57) | MgSO4 group | -0.2 | -0.55 | 0.15 | 0.259 |
|  |  |  |  |  | Initial MAP above cut-off | 0.04 | 0.02 | 0.05 | <0.001 |
| Imputation 3 |  |  |  |  |  |  |  |  |  |
| Median (range) | 12 | 1.59 (1.35 - 2.18) | 33 | 1.62 (1.39 - 1.86) | Intercept | 1.45 | 1.15 | 1.74 | <0.001 |
| Mean (SD) | 12 | 1.63 (0.73) | 33 | 1.54 (0.57) | MgSO4 group | -0.2 | -0.53 | 0.14 | 0.258 |
|  |  |  |  |  | Initial MAP above cut-off | 0.03 | 0.02 | 0.05 | <0.001 |
| Imputation 4 |  |  |  |  |  |  |  |  |  |
| Median (range) | 11 | 1.65 (1.23 - 2.18) | 33 | 1.62 (1.39 - 1.86) | Intercept | 1.32 | 1 | 1.63 | <0.001 |
| Mean (SD) | 11 | 1.61 (0.76) | 33 | 1.54 (0.57) | MgSO4 group | -0.06 | -0.4 | 0.28 | 0.725 |
|  |  |  |  |  | Initial MAP above cut-off | 0.03 | 0.02 | 0.05 | <0.001 |
| Imputation 5 |  |  |  |  |  |  |  |  |  |
| Median (range) | 9 | 1.75 (1.40 - 2.17) | 33 | 1.62 (1.39 - 1.86) | Intercept | 1.47 | 1.12 | 1.82 | <0.001 |
| Mean (SD) | 9 | 1.71 (0.80) | 33 | 1.54 (0.57) | MgSO4 group | -0.2 | -0.57 | 0.18 | 0.303 |
|  |  |  |  |  | Initial MAP above cut-off | 0.03 | 0.02 | 0.05 | <0.001 |
| Imputation 6 |  |  |  |  |  |  |  |  |  |
| Median (range) | 10 | 1.69 (1.42 - 2.17) | 33 | 1.62 (1.39 - 1.86) | Intercept | 1.53 | 1.23 | 1.83 | <0.001 |
| Mean (SD) | 10 | 1.79 (0.55) | 33 | 1.54 (0.57) | MgSO4 group | -0.25 | -0.57 | 0.07 | 0.14 |
|  |  |  |  |  | Initial MAP above cut-off | 0.03 | 0.02 | 0.04 | <0.001 |
| Imputation 7 |  |  |  |  |  |  |  |  |  |
| Median (range) | 11 | 1.65 (1.44 - 2.17) | 33 | 1.62 (1.39 - 1.86) | Intercept | 1.5 | 1.2 | 1.79 | <0.001 |
| Mean (SD) | 11 | 1.70 (0.71) | 33 | 1.54 (0.57) | MgSO4 group | -0.24 | -0.58 | 0.09 | 0.159 |
|  |  |  |  |  | Initial MAP above cut-off | 0.03 | 0.02 | 0.05 | <0.001 |
| Imputation 8 |  |  |  |  |  |  |  |  |  |
| Median (range) | 12 | 1.51 (1.16 - 2.16) | 33 | 1.62 (1.39 - 1.86) | Intercept | 1.35 | 1.05 | 1.64 | <0.001 |
| Mean (SD) | 12 | 1.58 (0.73) | 33 | 1.54 (0.57) | MgSO4 group | -0.09 | -0.42 | 0.24 | 0.589 |
|  |  |  |  |  | Initial MAP above cut-off | 0.03 | 0.02 | 0.05 | <0.001 |
| Imputation 9 |  |  |  |  |  |  |  |  |  |
| Median (range) | 12 | 1.61 (1.34 - 2.19) | 33 | 1.62 (1.39 - 1.86) | Intercept | 1.34 | 1.04 | 1.65 | <0.001 |
| Mean (SD) | 12 | 1.64 (0.71) | 33 | 1.54 (0.57) | MgSO4 group | -0.08 | -0.41 | 0.24 | 0.618 |
|  |  |  |  |  | Initial MAP above cut-off | 0.03 | 0.02 | 0.05 | <0.001 |
| Imputation 10 |  |  |  |  |  |  |  |  |  |
| Median (range) | 10 | 1.70 (1.42 - 2.17) | 33 | 1.62 (1.39 - 1.86) | Intercept | 1.53 | 1.23 | 1.82 | <0.001 |
| Mean (SD) | 10 | 1.75 (0.63) | 33 | 1.54 (0.57) | MgSO4 group | -0.27 | -0.59 | 0.05 | 0.11 |
|  |  |  |  |  | Initial MAP above cut-off | 0.03 | 0.02 | 0.05 | <0.001 |
| Imputation 11 |  |  |  |  |  |  |  |  |  |
| Median (range) | 12 | 1.56 (1.25 - 2.16) | 33 | 1.62 (1.39 - 1.86) | Intercept | 1.34 | 1.04 | 1.64 | <0.001 |
| Mean (SD) | 12 | 1.60 (0.72) | 33 | 1.54 (0.57) | MgSO4 group | -0.08 | -0.41 | 0.25 | 0.644 |
|  |  |  |  |  | Initial MAP above cut-off | 0.03 | 0.02 | 0.05 | <0.001 |
| Imputation 12 |  |  |  |  |  |  |  |  |  |
| Median (range) | 12 | 1.58 (1.26 - 2.16) | 33 | 1.62 (1.39 - 1.86) | Intercept | 1.43 | 1.13 | 1.73 | <0.001 |
| Mean (SD) | 12 | 1.61 (0.73) | 33 | 1.54 (0.57) | MgSO4 group | -0.16 | -0.51 | 0.18 | 0.357 |
|  |  |  |  |  | Initial MAP above cut-off | 0.03 | 0.02 | 0.05 | <0.001 |
| Imputation 13 |  |  |  |  |  |  |  |  |  |
| Median (range) | 10 | 1.70 (1.38 - 2.21) | 33 | 1.62 (1.39 - 1.86) | Intercept | 1.57 | 1.29 | 1.86 | <0.001 |
| Mean (SD) | 10 | 1.79 (0.56) | 33 | 1.54 (0.57) | MgSO4 group | -0.31 | -0.63 | 0.01 | 0.062 |
|  |  |  |  |  | Initial MAP above cut-off | 0.03 | 0.02 | 0.05 | <0.001 |
| Imputation 14 |  |  |  |  |  |  |  |  |  |
| Median (range) | 12 | 1.56 (1.32 - 2.19) | 33 | 1.62 (1.39 - 1.86) | Intercept | 1.31 | 1.01 | 1.6 | <0.001 |
| Mean (SD) | 12 | 1.61 (0.72) | 33 | 1.54 (0.57) | MgSO4 group | -0.06 | -0.38 | 0.26 | 0.704 |
|  |  |  |  |  | Initial MAP above cut-off | 0.04 | 0.02 | 0.05 | <0.001 |
| Imputation 15 |  |  |  |  |  |  |  |  |  |
| Median (range) | 12 | 1.53 (1.38 - 2.16) | 33 | 1.62 (1.39 - 1.86) | Intercept | 1.47 | 1.18 | 1.76 | <0.001 |
| Mean (SD) | 12 | 1.64 (0.70) | 33 | 1.54 (0.57) | MgSO4 group | -0.21 | -0.55 | 0.12 | 0.216 |
|  |  |  |  |  | Initial MAP above cut-off | 0.03 | 0.02 | 0.05 | <0.001 |
| Imputation 16 |  |  |  |  |  |  |  |  |  |
| Median (range) | 12 | 1.58 (1.35 - 2.18) | 33 | 1.62 (1.39 - 1.86) | Intercept | 1.41 | 1.12 | 1.69 | <0.001 |
| Mean (SD) | 12 | 1.62 (0.72) | 33 | 1.54 (0.57) | MgSO4 group | -0.15 | -0.47 | 0.17 | 0.367 |
|  |  |  |  |  | Initial MAP above cut-off | 0.03 | 0.02 | 0.05 | <0.001 |
| Imputation 17 |  |  |  |  |  |  |  |  |  |
| Median (range) | 10 | 1.70 (1.38 - 2.20) | 33 | 1.62 (1.39 - 1.86) | Intercept | 1.5 | 1.21 | 1.79 | <0.001 |
| Mean (SD) | 10 | 1.79 (0.56) | 33 | 1.54 (0.57) | MgSO4 group | -0.23 | -0.54 | 0.08 | 0.149 |
|  |  |  |  |  | Initial MAP above cut-off | 0.03 | 0.02 | 0.05 | <0.001 |
| Imputation 18 |  |  |  |  |  |  |  |  |  |
| Median (range) | 10 | 1.69 (1.38 - 2.17) | 33 | 1.62 (1.39 - 1.86) | Intercept | 1.44 | 1.12 | 1.76 | <0.001 |
| Mean (SD) | 10 | 1.63 (0.82) | 33 | 1.54 (0.57) | MgSO4 group | -0.2 | -0.55 | 0.16 | 0.294 |
|  |  |  |  |  | Initial MAP above cut-off | 0.04 | 0.02 | 0.05 | <0.001 |
| Imputation 19 |  |  |  |  |  |  |  |  |  |
| Median (range) | 12 | 1.61 (1.34 - 2.18) | 33 | 1.62 (1.39 - 1.86) | Intercept | 1.41 | 1.12 | 1.71 | <0.001 |
| Mean (SD) | 12 | 1.63 (0.72) | 33 | 1.54 (0.57) | MgSO4 group | -0.16 | -0.49 | 0.17 | 0.344 |
|  |  |  |  |  | Initial MAP above cut-off | 0.03 | 0.02 | 0.05 | <0.001 |
| Imputation 20 |  |  |  |  |  |  |  |  |  |
| Median (range) | 12 | 1.58 (1.40 - 2.17) | 33 | 1.62 (1.39 - 1.86) | Intercept | 1.46 | 1.17 | 1.74 | <0.001 |
| Mean (SD) | 12 | 1.64 (0.71) | 33 | 1.54 (0.57) | MgSO4 group | -0.21 | -0.53 | 0.12 | 0.218 |
|  |  |  |  |  | Initial MAP above cut-off | 0.03 | 0.02 | 0.05 | <0.001 |
