# Supplementary material for: Assessing the efficacy and safety of magnesium sulfate for management of autonomic nervous system dysregulation in Vietnamese children with severe hand foot and mouth disease
Source: BMC Infect Dis. 2019 Aug 22;19:737. doi: 10.1186/s12879-019-4356-x (PMC6704683; doi:10.1186/s12879-019-4356-x)
Supplement: Supplementary file 6 — Figure S1. The evolution of plasma and urine catecholamine levels over time in the two study arms. (DOCX 731 kb) [file 12879_2019_4356_MOESM6_ESM.docx]

Additional file 6: Figure S1: The evolution of plasma and urine catecholamine levels over time in the two study arms.


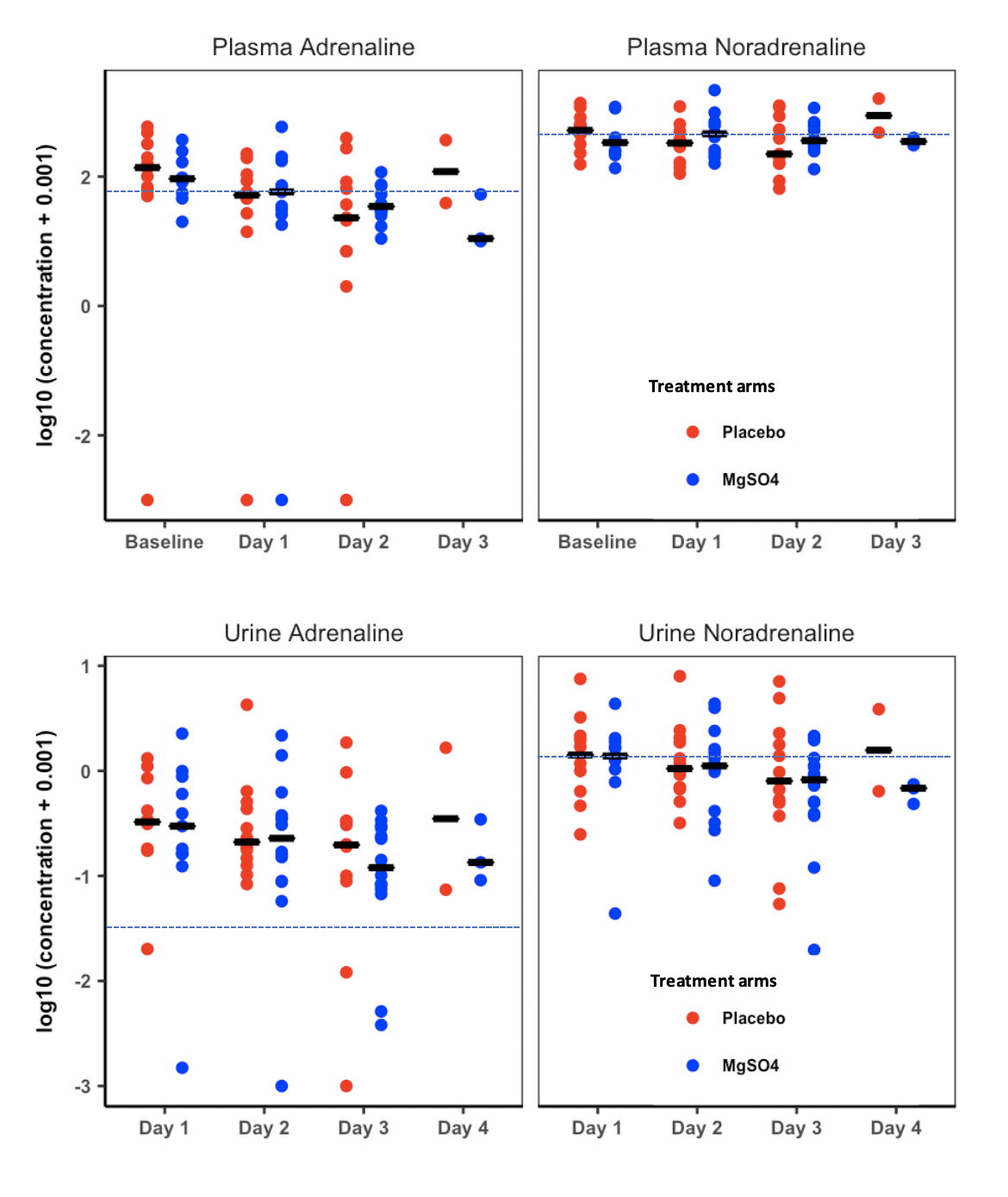


Dotted blue lines represent the upper limit of normal in children for the respective measurements.

Since the distribution of the catecholamine levels was skewed the data were log-transformed. Since levels of some samples were below the assay sensitivity level, 0.001 was added to each result to avoid a final result of infinity when taking the log10 value of 0.
